# Artificial intelligence for automated detection and measurements of carpal instability signs on conventional radiographs

**DOI:** 10.1007/s00330-024-10744-1

**Published:** 2024-04-18

**Authors:** Nils Hendrix, Ward Hendrix, Bas Maresch, Job van Amersfoort, Tineke Oosterveld-Bonsma, Stephanie Kolderman, Myrthe Vestering, Stephanie Zielinski, Karlijn Rutten, Jan Dammeier, Lee-Ling Sharon Ong, Bram van Ginneken, Matthieu Rutten

**Affiliations:** 1 Department of Medical Imaging, Radboud University Medical Center, Geert Grooteplein Zuid 10, 6525 GA Nijmegen, The Netherlands; 2 Jheronimus Academy of Data Science, Sint Janssingel 92, 5211 DA ‘s-Hertogenbosch, The Netherlands; 3 Department of Radiology, Jeroen Bosch Ziekenhuis, Henri Dunantstraat 1, 5223 GZ ‘s-Hertogenbosch, The Netherlands; 4 Department of Radiology, Ziekenhuis Gelderse Vallei, Willy Brandtlaan 10, 6717 RP Ede, The Netherlands; 5 Department of Surgery, Ziekenhuis Gelderse Vallei, Willy Brandtlaan 10, 6717 RP Ede, The Netherlands; 6 Cognitive Science and Artificial Intelligence Department, Tilburg University, Warandelaan 2, 5037 AB Tilburg, The Netherlands

**Keywords:** Wrist, Radiography, Artificial intelligence

## Abstract

**Objectives:**

To develop and validate an artificial intelligence (AI) system for measuring and detecting signs of carpal instability on conventional radiographs.

**Materials and methods:**

Two case-control datasets of hand and wrist radiographs were retrospectively acquired at three hospitals (hospitals A, B, and C). Dataset 1 (2178 radiographs from 1993 patients, hospitals A and B, 2018–2019) was used for developing an AI system for measuring scapholunate (SL) joint distances, SL and capitolunate (CL) angles, and carpal arc interruptions. Dataset 2 (481 radiographs from 217 patients, hospital C, 2017–2021) was used for testing, and with a subsample (174 radiographs from 87 patients), an observer study was conducted to compare its performance to five clinicians. Evaluation metrics included mean absolute error (MAE), sensitivity, and specificity.

**Results:**

Dataset 2 included 258 SL distances, 189 SL angles, 191 CL angles, and 217 carpal arc labels obtained from 217 patients (mean age, 51 years ± 23 [standard deviation]; 133 women). The MAE in measuring SL distances, SL angles, and CL angles was respectively 0.65 mm (95%CI: 0.59, 0.72), 7.9 degrees (95%CI: 7.0, 8.9), and 5.9 degrees (95%CI: 5.2, 6.6). The sensitivity and specificity for detecting arc interruptions were 83% (95%CI: 74, 91) and 64% (95%CI: 56, 71). The measurements were largely comparable to those of the clinicians, while arc interruption detections were more accurate than those of most clinicians.

**Conclusion:**

This study demonstrates that a newly developed automated AI system accurately measures and detects signs of carpal instability on conventional radiographs.

**Clinical relevance statement:**

This system has the potential to improve detections of carpal arc interruptions and could be a promising tool for supporting clinicians in detecting carpal instability.

## Key Points

- *Artificial intelligence can accurately measure and detect signs of carpal instability on conventional radiographs.*
- *It measures scapholunate distances, scapholunate and capitolunate angles, and detects carpal arc interruptions. The measurement accuracy was largely comparable to five clinicians with different specialties, and the arc interruption detections were more accurate than most clinicians.*
- *All measurements and detections are explainable, and a new heat map technique was developed for visualizing the degree and location of carpal arc interruptions in the image.*

**Graphical Abstract:**

## Graphical abstract

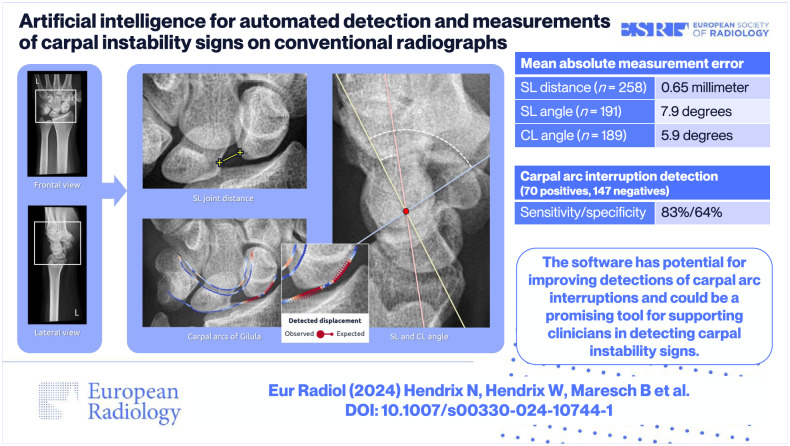

## Introduction

Carpal instability exists when the carpal bones cannot maintain their normal alignment under physiologic loads and movements [1]. Most forms of carpal instability are caused by acute trauma, such as ligament ruptures and displaced fractures. Nontraumatic causes are less common and include inflammatory arthritis, infections, and congenital disorders. While the true prevalence of carpal instability remains unknown, traumatic ligament injuries have been found to frequently co-occur with acute wrist fractures. In studies using surgically verified data (i.e., wrist arthroscopy), they were reported to be present in 34% of all scaphoid fractures [2] and 13–64% of all distal radius fractures [3–5]. Scapholunate (SL) ligament tears were commonly identified in both types of fractures. It is important to identify these injuries at an early stage, as they could lead to SL dissociation and SL advanced collapse (SLAC) if untreated [6]. It has been reported that signs of carpal instability co-occurring with acute wrist fractures are frequently overlooked on conventional radiographs [7–9]. As conventional radiography is usually the first imaging modality of choice after suspected wrist trauma [10], it can be worthwhile to focus research efforts on detecting signs of carpal instability on conventional radiographs.

To identify carpal instability on conventional radiography, it is recommended to evaluate for widened intercarpal joint distances and abnormal carpal angles [1, 11]. Widening of the SL joint can be indicative of traumatic tears of the SL ligament. In addition, disruptions of the carpal arc alignment are useful radiological features for identifying this condition and other causes of carpal instability [12]. However, carpal measurements have been shown to be subject to human variation and error [13–15] and may be unfamiliar to clinicians other than musculoskeletal (MSK) radiologists and (hand) surgeons. Therefore, a reliable and automated system to measure and detect signs of carpal stability could prove to be a valuable tool in daily clinical practice.

In recent years, artificial intelligence (AI) software has shown high performance in automating various tasks in the field of musculoskeletal radiology [16–19]. These tasks range from quantifications, such as bone age assessments and body composition measurements, to lesion detections, such as bone fractures and tumors. In light of these advances, we propose an explainable and unified framework for automatically measuring and detecting a wide variety of carpal instabilities. To limit the scope of the study, we demonstrate the value of this framework for assessments of the SL joint distance, SL and capitolunate (CL) angle, and carpal alignment. The purpose of this study was twofold: (a) to develop and validate an AI system that can accurately measure and detect signs of carpal instability; and (b) to assess how this system compares to clinicians with various specialties in measuring and detecting signs of carpal instability on conventional radiographs.

## Materials and Methods

### Datasets

This retrospective study was approved by the local institutional boards of the Radboud University Medical Center (Radboudumc), Jeroen Bosch Hospital (JBZ), and Hospital Gelderse Vallei (ZGV) in The Netherlands. Informed written consent was waived, and data collection, anonymization, and storage were performed in accordance with local guidelines. Two datasets of hand, wrist, and scaphoid radiographs were prepared for training and evaluating the AI system. An overview of the characteristics of these datasets is provided in Table 1. Additional imaging parameters are provided in Appendix E1 (online).

**Table 1:** Details of the experimental datasets

Variable	Dataset 1	Dataset 2	
		Total	Observer study set
Task	Train AI system	Test AI system	Compare AI system
No. of patients	1993	217	87
Sex			
Male	890 (44.7%)	84 (38.7%)	35 (40.2%)
Female	1103 (55.3%)	133 (61.3%)	52 (59.8%)
Age			
All	45 ± 23	51 ± 23	56 ± 22
Male	40 ± 22	44 ± 21	50 ± 18
Female	49 ± 24	55 ± 24	59 ± 23
Number of radiographs	2178	481	174
Radiograph Location			
Hand	778 (35.7%)	46 (9.6%)	4 (2.3%)
Wrist	1265 (58.1%)	395 (82.1%)	162 (93.1%)
Scaphoid	135 (6.2%)	40 (8.3%)	8 (4.6%)
View			
Neutral AP/PA	775 (35.6%)	210 (43.7%)	70 (40.2%)
Clenched fist AP/PA	17 (0.8%)	48 (10.0%)	17 (9.8%)
Ulnar-deviated AP/PA	29 (1.3%)	3 (0.6%)	0 (0%)
Oblique	286 (13.1%)	29 (6.0%)	0 (0%)
Lateral	1071 (49.2%)	191 (39.7%)	87 (45.1%)
Number studies	2093	217	87
Carpal stability status^a^			
Abnormal SL distance	NA	65	39
Abnormal SL angle	NA	98	38
Abnormal CL angle	NA	20	20
Interrupted carpal arcs	NA	70	44
Normal measurements	NA	83	28
Fracture status^b^			
Wrist fracture^c^	NA	66	31
Metacarpal fracture	NA	7	2
No fracture	NA	145	54
Source(s)	Radboudumc, JBZ	ZGV	ZGV
Period	01/2018–04/2019	01/2017–12/2021	01/2017–11/2021

Percentages with respect to the total dataset size are in parentheses. Rounding errors were resolved using the largest remainder method. ^a^The carpal stability status was only determined for a subset of dataset 1 for development purposes (see Appendix E2 [online] for more details). ^b^The fracture status according to the original radiology reports. ^c^Includes the carpal bones and the distal radius and the ulna.

*NA* not applicable, *Radboudumc* Radboud University Medical Center, *ZGV* Hospital Gelderse Vallei

#### Dataset 1

Dataset 1 consisted of 2178 radiographs (1993 patients) that were obtained at Radboudumc and JBZ in 2018–2019. It consisted of an equal portion of frontal view (including neutral, ulnar-deviated, clenched fist anterior-posterior [AP] or posterior-anterior [PA], and oblique) and lateral view radiographs. Radiographs were excluded when the outlines of the carpal bones could not be delineated due to metal implants and casts or excessive degeneration, fusion, or destruction of bones. The latter conditions can occur due to high-energy trauma or chronic disease, such as end-stage rheumatoid arthritis. The annotation protocol and a flow chart of the training data selection (dataset 1) are respectively provided in Appendices E2 and E3 (online).

#### Dataset 2

**Fig. 1:**
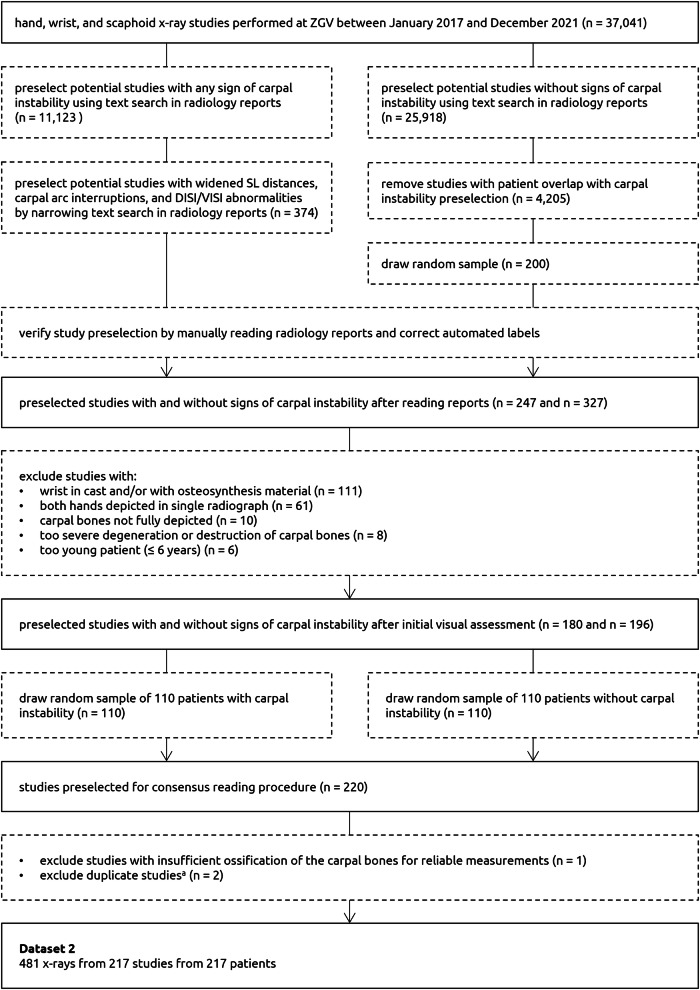
Flowchart for the inclusion and exclusion of studies in dataset 2 (test set). The number of studies at each step is denoted with *n*. Studies were preselected from the text search results in random order. ^a^Based on the study metadata (e.g., study date, patient demographics, report), these studies were found to be duplicates of studies that were already included

Dataset 2 consisted of 481 radiographs (217 patients; one study per patient) obtained at ZGV in 2017–2021. This dataset served for evaluating the automated measurements of the AI system. The studies were preselected based on the original radiology reports to balance the number of patients with and without signs of carpal instability. A flow chart of the test data selection (dataset 2) is shown in Fig. 1. Stricter exclusion criteria were applied as compared to dataset 1 by excluding radiographs with (a) any overprojection caused by metal implants and casts, (b) no neutral (relaxed) lateral wrist position, (c) non or partially ossified and developed carpus (in children). The first criterion was added to maximize the reliability of the measurements. The latter two criteria were minimally required for performing the measurements (see “Measurement Definitions” section). The annotation protocol is described in the section “Reference Standard”.

A subset of dataset 2 was used for conducting the subsequent observer study (see “Observer study” section). It consisted of 87 studies (193 radiographs from 87 patients). The subset was balanced by including at least 20 studies for each of the following conditions: abnormal CL angle, abnormal SL angle, abnormal SL joint distance, interrupted carpal arcs, and no abnormal measurements. The distribution of distance and angle measurement values for dataset 2 (full and subset) is provided in Appendix E4.

### Measurement definitions

The measurements investigated in this study were defined according to the literature [20]. The SL joint distance was measured on the mid-points of the scaphoid-lunate facet on the AP/PA view. Dornberger et al [21] showed that thresholds of 3.0 and 3.7 mm on respectively neutral and ulnar-deviated views (Stecher’s projections) were optimal. SL joint distances exceeding these thresholds were considered abnormal in this study. For children (6–14 years old), the thresholds were set to the upper limit of the normal values per age group, as reported by Kaawach et al [22].

The SL and CL angles were defined as the angles between the long axis of the scaphoid or capitate and the mid-plane axis of the lunate on the lateral radiographic view [11]. In a neutral wrist position, the SL angle should be between 30 and 60 degrees, and the CL angle should be less than 30 degrees. Values exceeding these reference values were considered abnormal. The same thresholds were used for children and adolescents.

The carpal arcs were defined on the neutral AP/PA view as proposed by Gilula [12]. The arcs were considered interrupted or abnormal in cases of carpal dislocations, carpal collapses, or dislocated carpal fractures. Dissociations causing the arcs only to lengthen were not considered interruptions unless bones subluxated proximally into the widened joint spaces. This also applied to normal anatomical variants (i.e., shortened triquetrum or bi-lobed/type II lunate morphology [23]) or narrowed joint spaces.

### Reference standard

The reference standard was determined by a consensus reading of two experienced MSK radiologists (M.R. and B.M., with 27 and 26 years of experience, respectively). They independently annotated the test dataset (dataset 2) and resolved any discrepancies using the consensus reading procedure as described in Appendix E5 (online).

### AI pipeline

**Fig. 2:**
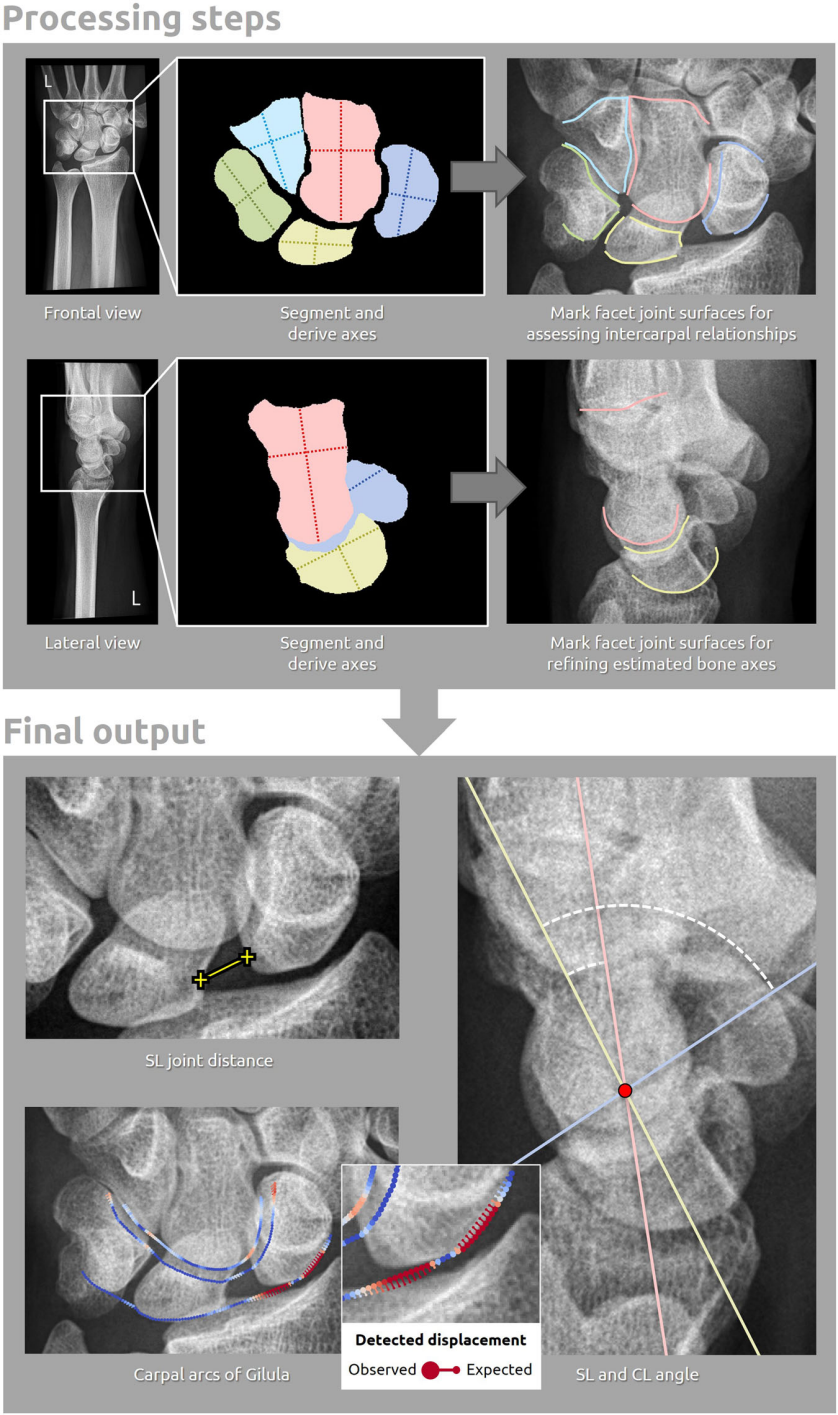
Overview of the (AI) pipeline for measuring and detecting signs of carpal instability in frontal and lateral view radiographs. The spatial and geometric properties of the relevant carpal bones are determined by segmentation and are then used to identify the articular facet joint surfaces. Based on the obtained bone surfaces and angles, the carpal instability measurements and detections can be conducted. The generated carpal arcs are visualized as color-coded points (*n* = 100) that form an easily interpretable heatmap. The warmer colors indicate significant deviations from the reconstructed hypothetical normal arcs (expressed as z-scores). These deviations or distances are shown by the small tails attached to the points (displacement vectors). More information can be found in Appendix E6 (online)

The pipeline of the AI system is summarized in Fig. 2. The system was designed to take a radiograph as input and to return the following outputs (depending on the provided view): (a) SL joint distance in millimeters, (b) SL angle in degrees, (c) CL angle in degrees, (d) polylines of the carpal arcs, and (e) markers of potential disruptions of the carpal arcs with an overall disruption score. The pipeline consisted of three general steps. Two convolutional neural networks (CNNs) first segmented the anterior and lateral sides of the carpals on the AP/PA view and lateral view, respectively. Next, the orientation (major/minor axis) and articular facet joint surfaces of the bones were determined using active appearance models (AAMs). Last, all measurements and subsequent detections from the articular surfaces and bone axes were automatically derived by the AI system. Interruptions of the carpal arcs were determined by comparing the observed and reconstructed hypothetical shape of the carpal arc polylines if noninterrupted (obtained from a point distribution model [PDM]). A newly developed heat map technique using vectors and color-coding visualizes the degree (z-score) and location of carpal arc interruptions in the original image (Fig. 2). The system is publicly available at https://grand-challenge.org/algorithms/, where it can be run in a web browser. A detailed description of the processing steps and training procedure is provided in Appendices E6 and E7 (online).

### Observer study

An observer study was conducted among five clinicians: an MSK radiologist (M.V.), non-MSK radiologist (S.K.), hand surgeon (S.Z., EBHS certified), junior doctor on general surgery (J.v.A.), and emergency (ER) doctor (T.O.-B.) with 14, 12, 16, 1, and 24 years of experience, respectively. The clinicians independently measured in the subset of database 2 the SL joint distance, SL and CL angle (derived from axes) as defined in the “Measurement Definitions” section. They indicated their confidence on a five-point Likert scale whether the carpal arcs were interrupted. The lowest and highest scores, respectively, indicated that the carpal arcs were definitely normal and abnormal, while the neutral option (uncertain, interruptions might be present or not present) was the cut-off point for the clinical decision for follow-up examination. The clinicians assessed all cases using the Cirrus Core Workstation on the web platform Grand Challenge (version 2022.07, 2022) [24] and had access to all radiographic views per patient.

### Statistical analysis

#### Evaluation metrics

The segmentation and landmark localization component of the AI system were separately evaluated on dataset 1. The evaluation details are provided in Appendix E8 (online). The whole AI system was evaluated on dataset 2. The accuracy of the measurements and generated carpal arcs were respectively evaluated using the mean absolute error (MAE) and mean Fréchet distance (MFD). The measurement agreement with the reference standard was evaluated using the bias and limits of agreement (LoA) obtained from a Bland-Altman plot analysis [25]. The ability to detect abnormal distances, angles, and carpal arc interruptions was evaluated using the following metrics: sensitivity, specificity, and area under the receiver operating characteristic (ROC) curve (AUC) (arc interruptions only). For evaluating carpal arc interruption detections, the detection threshold of the AI system was selected that maximized the Youden’s index. The Fréchet distance was calculated using the similarity measures Python library (version 0.7.0, 2023) [26]. The Bland-Altman plot analysis was conducted using the pyCompare Python library (version 1.5.4, 2022) [27]. The other metrics were calculated using the scikit-learn Python library (version 1.2.1, 2023) [28].

#### Significance tests

Stratified bootstrapping with 1000 iterations was applied for estimating 95% confidence intervals (CIs), except for the Bland-Altman plots that were calculated as described in [25]. Stratification was conducted by grouping data per 1 mm (distances), 10 degrees (angles), and binary labels (detections). Significance testing was performed with two-sided paired permutation tests with 1000 iterations using the MLxtend Python library (version 0.21.0, 2022) [29], except for the AUCs that were compared with DeLong tests [30] using the pyroc Python library (version 0.2.0, 2022) [31]. Differences with a *p* value smaller than 0.05 were considered significant.

## Results

### Test data characteristics

Five hundred and seventy-four studies with and without signs of carpal instability were preselected based on the radiology reports. After the initial visual assessment, 196 studies were excluded. These studies involved the following cases: wrist in cast and/or with osteosynthesis material (*n* = 111), both hands depicted in a single radiograph (*n* = 61), carpal bones not fully depicted (*n* = 10), too severe degeneration or destruction of the carpal bones (*n* = 8), too young patient (≤ 6 years old; *n* = 6). Cases with severe degeneration or destruction (e.g., SLAC, displaced fractures) were only excluded when the annotation was no longer possible due to disappeared articular surfaces (*n* = 6), excessive osteoporosis (*n* = 1), or complete isolated scaphoid dislocation (*n* = 1).

Next, from the 220 studies randomly sampled for the consensus reading procedure, two duplicate studies and one study examining a patient with insufficient ossification of the carpal bones were excluded. Three SL distance and SL angle measurements were respectively excluded due to insufficient visibility of the anterior side of the lunate and the lateral side of the scaphoid. This resulted into a final selection of 217 studies from 217 patients (mean age, 51 years ± 23 [standard deviation {SD}]; 133 women). As the studies contained multiple radiographic series, the measurements included 258 SL distances, 189 SL angles, 191 CL angles, and 217 sets of labelled carpal arcs. All studies selected for the observer study (subset of dataset 2) contained at least one AP/PA and lateral view radiograph (no excluded measurements), so that all measurements could be conducted by the clinicians.

### Evaluation of the AI system

#### Measurement and detection results

**Fig. 3:**
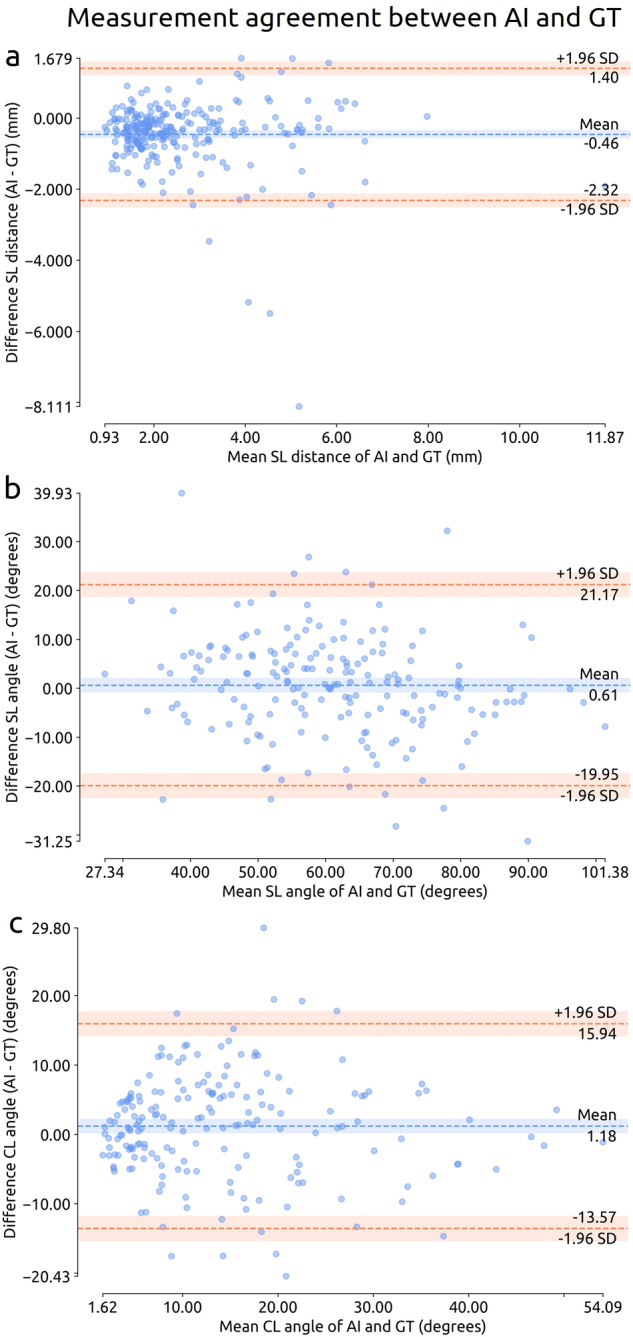
Bland-Altman plots of the measurement agreement between the AI system and the GT on the measurements of the SL distance (*n* = 258, see **a**), SL angle (*n* = 189, see **b**), and CL angle (*n* = 191, see **c**) in dataset 2. Each marker represents one paired measurement. The dashed lines represent the mean difference (blue) and LoA (orange). The shaded bands represent 95%CI

**Fig. 4:**
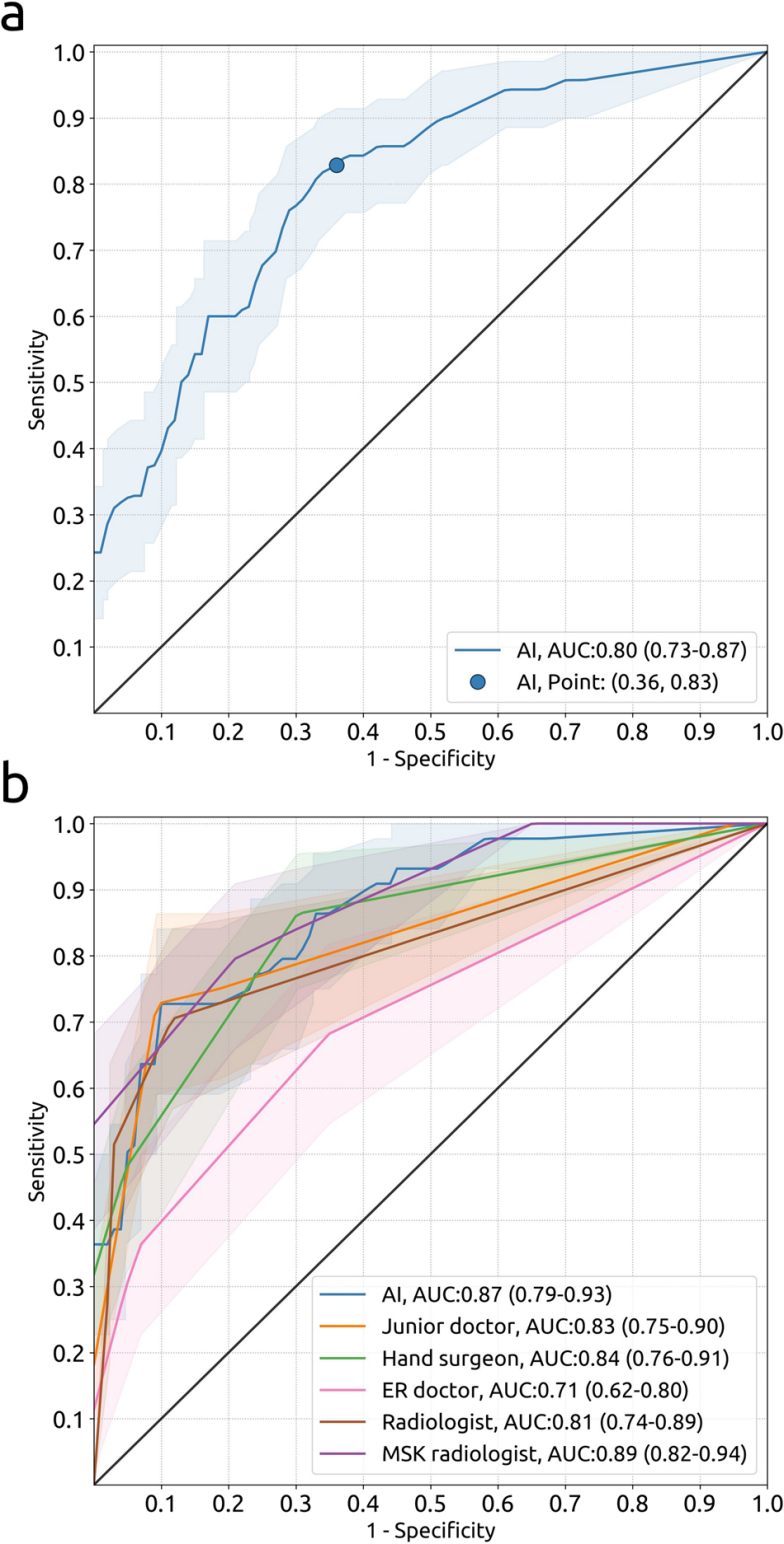
**a** ROC curve with the operating point of the carpal arc interruption detection results of the AI system on dataset 2 (70 positive cases, 147 negative cases). **b** ROC curves of the carpal arc interruption detection results of the AI system and those of the clinicians on the observer study subset (44 positive cases, 43 negative cases). Each case represents one study from one patient. The shaded bands represent 95%CIs. The black line represents no ability to discriminate between interrupted and non-interrupted arcs. AUC = area under the ROC curve

Table 2 presents the measurement error (MAE, bias, LoA, MFD) of the AI system for SL joint distances, SL and CL angles, and carpal arcs with their 95% CIs in dataset 2. The MAEs in measuring the SL joint distance, SL angle, and CL angle on the total dataset were 0.65 mm, 7.9 degrees, and 5.9 degrees, respectively. The corresponding Bland-Altman plots are shown in Fig. 3, and the detection results are included in Appendix E9. The MFDs in measuring the proximal, middle, and distal carpal arc on the total dataset were 1.34, 1.15, and 1.25 mm, respectively. The sensitivity, specificity, and AUC in detecting interruptions in the carpal arcs were 83% (95%CI: 74%, 91%), 64% (95%CI: 56%, 71%), and 0.80 (95%CI: 0.73, 0.87), respectively (detection threshold was set to 11%). The corresponding ROC curve with 95%CI bands is shown in Fig. 4a.

**Table 2:** Measurement results of the AI system on dataset 2

Measurement	Value	95%CI
SL distance (mm) (*n* = 258)		
MAE	0.65	(0.59, 0.72)
Bias	−0.46	(−0.58, −0.35)
Lower LoA	−2.32	(−2.52, −2.12)
Upper LoA	+1.40	(+1.20, +1.59)
SL angle (degrees) (*n* = 189)		
MAE	7.9	(7.0, 8.9)
Bias	+0.6	(−0.9, +2.1)
Lower LoA	−20.0	(−22.5, −17.4)
Upper LoA	+21.2	(+18.6, +23.7)
CL angle (degrees) (*n* = 191)		
MAE	5.9	(5.2, 6.6)
Bias	+1.2	(+0.1, +2.3)
Lower LoA	−13.6	(−15.4, −11.7)
Upper LoA	+15.9	(+14.1, +17.8)
Carpal arcs (mm) (*n* = 217)		
MFD proximal arc	1.34	(1.20, 1.49)
MFD middle arc	1.15	(1.02, 1.29)
MFD distal arc	1.25	(1.11, 1.41)

The bias (mean) and LoA are reported for the difference between the AI system and ground truth (GT) (AI – GT). The number of measurements is denoted with *n*. mm = millimeter.

#### Results of auxiliary system components

The carpal bone segmentation and landmark localization results are included in Appendices E10 and E11 (online).

### Comparison of the AI system to the clinicians

#### Measurement and detection results

Table 3 presents the measurement error (MAE, bias, LoA) of the AI system and clinicians for SL joint distances, SL and CL angles with their 95%CIs and *p* values. The corresponding Bland-Altman plots are provided in Appendix E12. Table 4 presents the detection performance (sensitivity, specificity, AUC) of the AI system and clinicians for interrupted carpal arcs with their 95%CIs and *p* values. The ROC curves for the carpal arc interruption detections are shown in Fig. 4b.

**Table 3:** Comparison of measurement results between the AI system and clinicians

Reader	SL distance measurement (*n* = 87)				
	MAE				
	Value	*p*	Bias	Lower LoA	Upper LoA
AI	0.62 (0.50, 0.73)		−0.37 (−0.56, −0.18)	−2.10 (−2.43, −1.78)	+1.36 (+1.03, +1.68)
Junior doctor	0.60 (0.50, 0.72)	0.89	−0.15 (−0.34, +0.03)	−1.87 (−2.19, −1.55)	+1.57 (+1.25, +1.89)
Hand surgeon	0.40 (0.33, 0.49)	0.003	−0.03 (−0.16, +0.09)	−1.17 (−1.38, −0.96)	+1.10 (+0.89, +1.31)
ER doctor	0.60 (0.50, 0.72)	0.88	+0.23 (+0.06, +0.40)	−1.32 (−1.61, −1.03)	+1.78 (+1.49, +2.07)
Radiologist	0.49 (0.38, 0.63)	0.21	+0.04 (−0.13, +0.21)	−1.51 (−1.80, −1.22)	+1.60 (+1.31, +1.89)
MSK radiologist	0.57 (0.47, 0.66)	0.49	+0.05 (−0.12, +0.22)	−1.52 (−1.81, −1.22)	+1.62 (+1.33, +1.92)

Reader	SL angle measurement (*n* = 87)				
	MAE				
	Value	*p*	Bias	Lower LoA	Upper LoA
AI	7.7 (6.7, 8.7)		+2.1 (+0.03, +4.2)	−17.2 (−20.8, −13.6)	+21.4 (+17.8, +25.0)
Junior doctor	9.0 (7.6, 10.4)	0.11	+4.6 (+2.3, +6.8)	−16.4 (−20.3, −12.5)	+25.5 (+21.6, +29.4)
Hand surgeon	7.7 (6.4, 9.0)	0.97	−4.3 (−6.2, −2.4)	−22.0 (−25.3, −18.7)	+13.4 (+10.1, +16.7)
ER doctor	10.9 (9.3, 12.7)	0.01	−8.5 (−11.0, −6.0)	−31.4 (−35.6, −27.1)	+14.3 (+10.1, +18.6)
Radiologist	9.8 (7.8, 12.0)	0.048	+3.1 (+0.2, +6.0)	−23.6 (−28.5, −18.6)	+29.7 (+24.8, +34.7)
MSK radiologist	6.9 (5.6, 8.3)	0.39	−1.5 (−3.5, +0.6)	−20.4 (−23.9, −16.8)	+17.4 (+13.9, +21.0)

Reader	CL angle measurement (*n* = 87)				
	MAE				
	Value	*p*	Bias	Lower LoA	Upper LoA
AI	6.0 (5.0, 7.0)		+0.6 (−1.1, +2.2)	−14.7 (−17.6, −11.9)	+15.9 (+13.0, +18.7)
Junior doctor	4.5 (3.8, 5.2)	0.03	−0.3 (−1.5, +0.9)	−11.4 (−13.4, −9.3)	+10.8 (+8.7, +12.8)
Hand surgeon	4.0 (3.3, 4.8)	0.01	+0.2 (−1.0, +1.4)	−10.8 (−12.9, −8.8)	+11.3 (+9.2, +13.4)
ER doctor	5.8 (4.7, 6.9)	0.82	−0.65 (−2.4, +1.1)	−17.1 (−20.1, −14.0)	+15.8 (+12.7, +18.9)
Radiologist	5.7 (4.7, 6.9)	0.73	+2.2 (+0.6, +3.8)	−12.6 (−15.4, −9.9)	+17.0 (+14.3, 19.8)
MSK radiologist	3.4 (2.8, 3.9)	< 0.001	+0.4 (−0.5, +1.3)	−8.1 (−9.7, −6.5)	+8.9 (+7.3, +10.5)

The bias (mean) and LoA are reported for the difference between the AI system or reader and the GT (AI/reader – GT). The number of measurements is denoted with *n*. 95%CIs are reported in parentheses. The *p* values refer to the differences in evaluation metrics with respect to the AI system.

*ER* emergency room.

**Table 4:** Comparison of carpal arc interruption detection results between the AI system and clinicians

Reader	Sensitivity (%)			Specificity (%)			AUC	
	Value	Frac	*p*	Value	Frac	*p*	Value	*p*
AI	73 (59, 86)	32/44		91 (81, 98)	39/43		0.87 (0.79, 0.93)	
Junior doctor	75 (61, 86)	33/44	> 0.99	81 (70, 91)	35/43	0.37	0.83 (0.75, 0.90)	0.35
Hand surgeon	48 (34, 64)	21/44	< 0.001	95 (88, 100)	41/43	0.69	0.84 (0.76, 0.91)	0.50
ER doctor	36 (23, 50)	16/44	< 0.001	93 (84, 100)	40/43	> 0.99	0.71 (0.62, 0.80)	< 0.001
Radiologist	50 (36, 64)	22/44	0.01	98 (93, 100)	42/43	0.25	0.81 (0.74, 0.89)	0.18
MSK radiologist	84 (73, 93)	37/44	0.25	70 (56, 84)	30/43	0.01	0.89 (0.82, 0.94)	0.65

95%CIs are reported in parentheses. The *p* values refer to the differences in evaluation metrics with respect to the AI system. “Fraction” has been abbreviated to “Frac”. The carpal arc interruption detection threshold of the AI system was set to 11%.

#### Failure case analysis

**Fig. 5:**
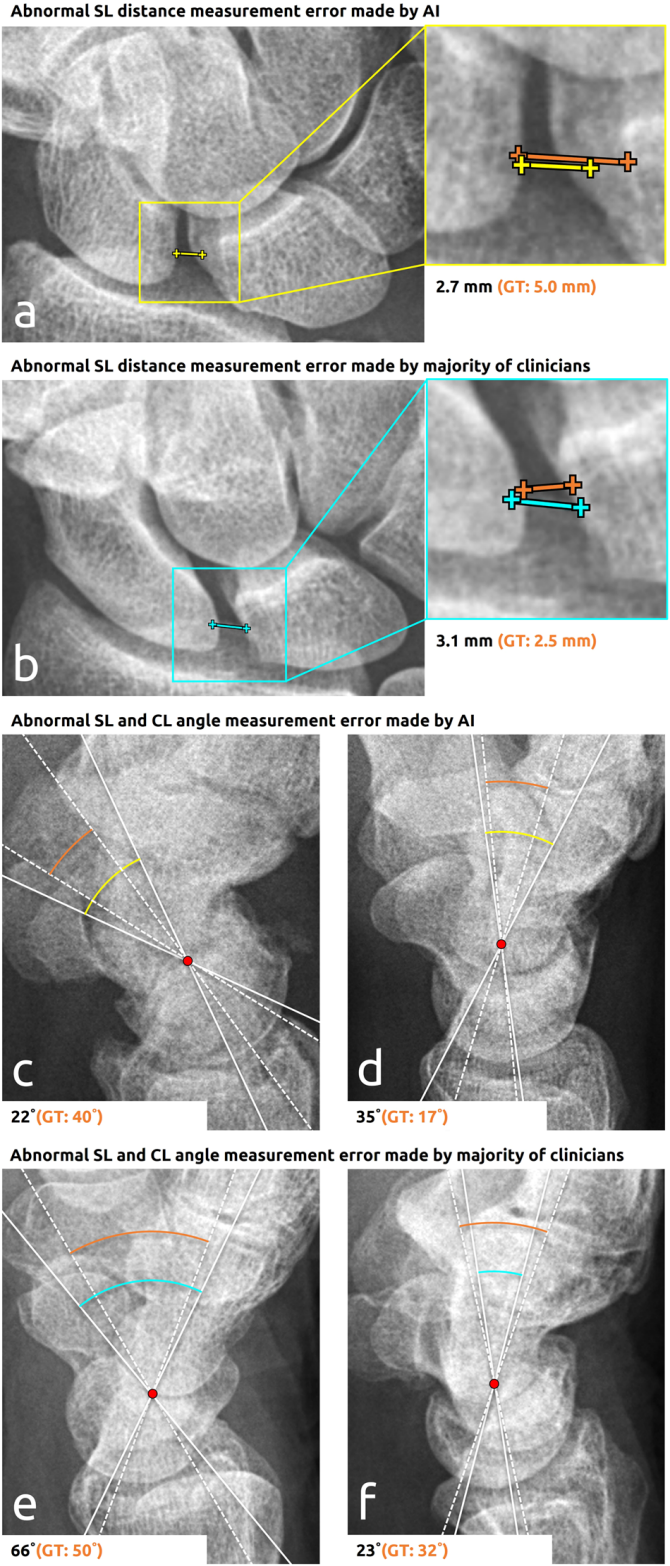
Example measurement and subsequent detection errors of abnormal SL joint distances, SL angles, and CL angles made by the AI system and clinicians. The lines in yellow, cyan, and orange, respectively, represent the AI, clinicians, and GT measurements. The axes of the angle measurements are shown in white with a dense pattern (AI or clinicians) and a dashed pattern (GT). The start and end coordinates of the lines corresponding to clinicians and GT have been averaged for this figure. The measurement value and corresponding GT are provided below each panel. **a** 77-year-old male with a widened SL joint distance (> 3 mm). **b** 37-year-old male with a normal SL joint distance. **c** 36-year-old male female with a normal SL angle. **d** 37-year-old male with a normal CL angle. **e** 74-year-old female with a normal SL angle. **f** 68-year-old female with an abnormal CL angle (> 30 degrees)

**Fig. 6:**
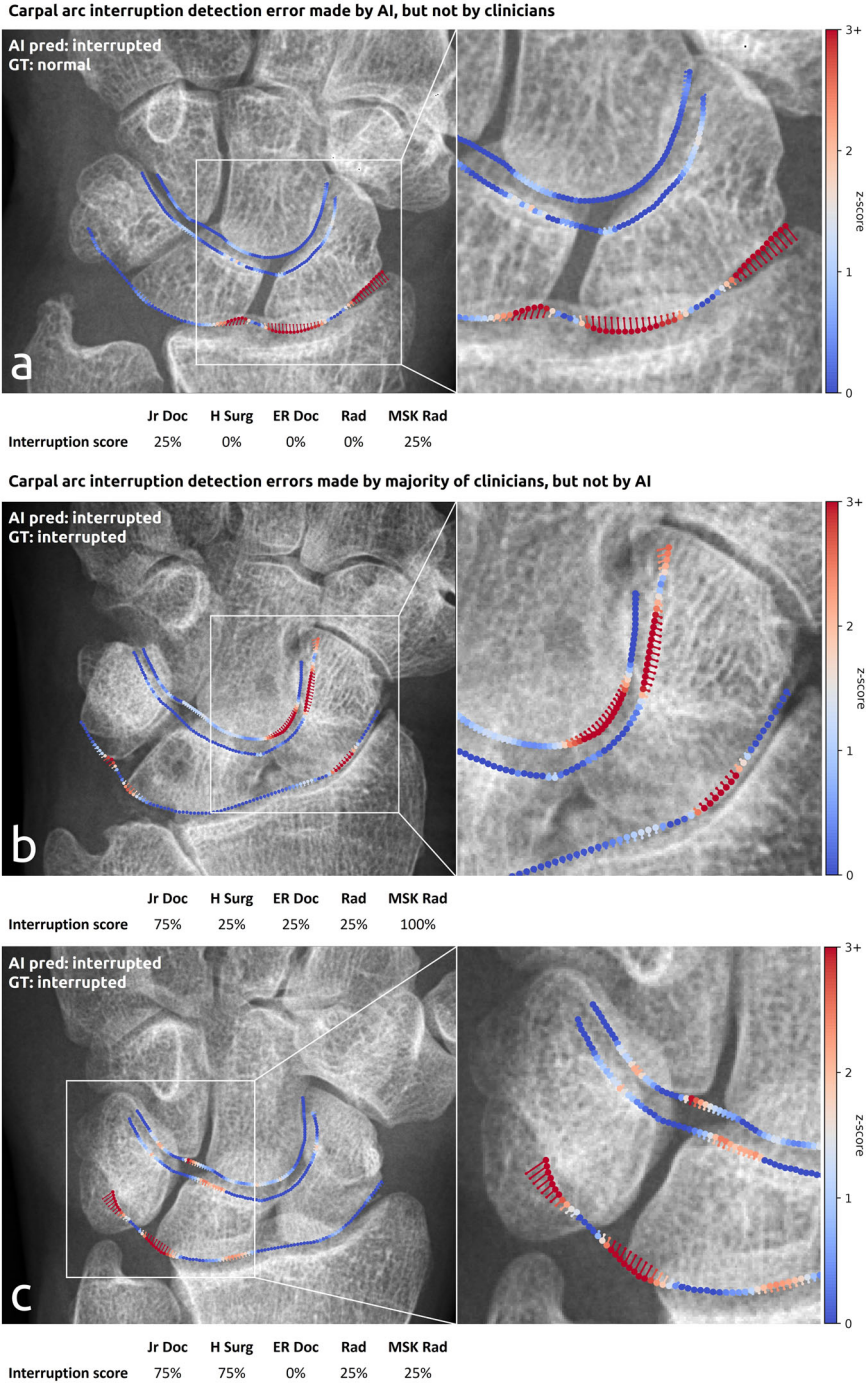
Example detection errors of carpal arc interruptions made by the AI system (**a**) and clinicians (**b** and **c**). The AI prediction and GT label are shown in the upper left corner of each image. The interruption scores of the clinicians are shown below each image (ranging from 0% [no interruption] to 100% [interruption]). The carpal arcs generated by the AI system are overlaid as color-coded points on the original image. The points correspond to z-scores: the higher the z-score, the more abnormal and hence indicative the point is of an interruption (see more information in Appendix E6 [online]). The deviations from the hypothetical normal shape of the carpal arcs are shown by the small tails attached to the points (displacement vectors). **a** 53-year-old female with a distal radius fracture and slight narrowing of the radiocarpal joint but normal carpal alignment. **b** 82-year-old male with the capitate subluxating proximally into the direction of a widened SL joint space. **c** 26-year-old male with slight angulation of the lunate accompanied by a widened SL joint space and semiacute scaphoid fracture. ER Doc = emergency doctor, H Surg = hand surgeon, Jr Doc = junior doctor, MSK Rad = musculoskeletal radiologist, Rad = radiologist

A qualitative analysis of the failure cases of the AI system and clinicians showed that the AI system made 13 measurement and subsequent detection errors that none of the clinicians made (from a total of 52 errors [AI]: 4/12 [SL distance], 5/16 [SL angle], 3/8 [CL angle], 1/16 [carpal arcs]). Example failure cases corresponding to the SL distances, SL and CL angles are shown in Fig. 5, and those corresponding to the carpal arcs are shown in Fig. 6. In four abnormal SL distance detection errors (false negatives), the segmentation of the scaphoid or lunate was elongated into the widened SL joint space (*n* = 2) or the measurement was carried out on the nonanterior side of the lunate (*n* = 2). In the former failure cases, displaced fracture parts of the distal radius moved into the SL joint space. In five abnormal SL angle detection errors (four false positives, one false negative), the lunate axis significantly deviated due to prediction errors of the midpoint on the lunate-radius facet. In the three CL angle detection errors (two false positives, one false negative), the lunate axis (*n* = 3) and capitate axis (*n* = 2) significantly deviated due to prediction errors of the midpoint on the lunate-radius, lunate-capitate, or capitate-metacarpal III facet. In the abnormal angle detection failure cases, the lunate was either significantly rounded or fractured (with displacement), or there was substantial overprojection on the lunate and capitate (from the other bones). In one carpal arc interruption detection error (false positive), the proximal carpal arc had a nonrelevant interruption, as the proximal contour of the lunate diverged from the radius, and the scaphoid was slightly tilted.

There were 20 measurement and subsequent detection errors made by the majority of clinicians that were not made by the AI system (from a total of 52 errors [clinicians]: 3/8 [SL distance], 6/16 [SL angle], 2/5 [CL angle], 9/23 [carpal arcs]). Example failure cases corresponding to the SL distances, SL and CL angles are shown in Fig. 5, and those corresponding to the carpal arcs are shown in Fig. 6. In three widened SL distance detection errors (false positives), the clinicians disagreed on the location of the anterior side of the lunate (due to pseudo-delineated or irregular contour) (*n* = 2) or scaphoid (due to pseudo-overarching surface) (*n* = 1). In six abnormal SL angle detection errors (three false positives, three false negatives), the scaphoid axis deviated due to overprojection on the dorsal side, especially from the triquetrum. In two abnormal CL angle detection errors (one false positive, one false negative), the capitate axis deviated due to an asymmetrical bone shape. In nine carpal arc interruption detection errors (one false positive, eight false negatives), the capitate and lunate respectively slightly subluxated proximally toward the widened SL joint space (*n* = 5) and radial carpal joint (*n* = 3), or the clinicians disagreed on the congruence of the articular surfaces between the lunate and its surrounding bones (*n* = 1).

## Discussion

Measurements for carpal instability on conventional radiographs can be inconsistent between examiners and may be unfamiliar to less experienced clinicians. This multicenter study shows that AI driven measurements and detections of radiological signs of carpal instability are feasible at a clinically acceptable level. The MAEs in measuring SL distances, SL angles, and CL angles were 0.65 mm, 7.9 degrees, and 5.9 degrees, respectively. The sensitivity and specificity for detecting arc interruptions were 83 and 64%, respectively. The observer study shows that the AI system had a comparable accuracy to most clinicians in measuring SL distances and SL angles (equal or higher [*p* < 0.05] than, respectively, four and five clinicians). It had a lower accuracy in measuring the CL angle than most clinicians (*p* < 0.05 for three clinicians), but the difference was slight (MAE, 6.0 vs. 4.0 degrees [clinician average]). The AI system had a higher sensitivity than three clinicians at equal specificity in detecting carpal arc interruptions (sensitivity/specificity, 73%/91% vs. 45%/95% [clinician average], *p* < 0.05 [sensitivity] and *p* ≥ 0.05 [specificity]). To the best of our knowledge, only Keller et al [32] have investigated the application of AI for one of the tasks and also demonstrated that SL distance measurements can be accurately automated. Based on our findings, we expect that AI can potentially improve detections of signs of carpal instability and enable efficient screening without additional workload for clinicians.

The failure case analysis revealed that there were qualitative differences between the AI system and clinicians. It was found that 25% (13/52) of the detection errors of the system were not made by any clinician. However, conversely, the system did not make 38% (20/52) of the detection errors made by the majority of clinicians. Compared to the clinicians, the system displayed a slight disadvantage in measuring CL angles and carrying out measurements in patients with a displaced wrist fracture. Given the co-occurrence of ligament injuries with acute wrist fractures, this is a relevant finding that should be addressed in future research. Nevertheless, it did not result in a lower detection performance overall.

The AI system displayed an advantage over the clinicians in determining the scaphoid axis when overprojection was present and in detecting subtle arc interruptions due to subluxations. In this regard, there was no distinct difference between clinicians who could be considered more specialized (i.e., MSK radiologist, hand surgeon) and less specialized (i.e., junior doctor, ER doctor) in assessing carpal instability. This suggests that the system may have merit for both kinds of clinicians in these cases. However, it is important to note that generally the more specialized clinicians tended to have a lower measurement and detection error than the less specialized clinicians. Furthermore, while the system had a higher sensitivity for arc interruptions than the ER doctor, non-MSK radiologist, and hand surgeon, the difference in AUC was only significant for the ER doctor. This indicates that the confidence scores of the hand surgeon and radiologist were on the conservative side (i.e., the optimal threshold was lower than the neutral point on the Likert scale). For those clinicians, the system could potentially be beneficial in confirming suspicions of interrupted carpal arcs. Follow-up research with more clinicians per profession is required to confirm our findings.

This study had several limitations. First, the studies in the test dataset were collected in a case-control manner and were mainly (pre)selected based on the original radiology reports. Radiology reports were used for the selection due to the absence of reliable and specific diagnosis codes in the electronic health record (EHR) system. Subtle signs of carpal instability may not always have been reported, and this could have introduced selection bias, although we found that the test dataset contained a sufficient variety of measurement values. Second, cases with osteosynthesis material (e.g., metal plates, screws, k-wires) and casts were excluded in the test dataset to maximize the reliability of the measurements. We expect that this does not significantly affect the software performance as long as the joint spaces and surfaces are freely projected, but this should be investigated in future research. Third, we only focused on assessing the accuracy of the automated measurements and did not link the derived detections of carpal instability signs to the diagnosis. Carpal instability can be diagnosed with wrist arthroscopy or alternatively with MRI and CT arthrography. Nonetheless, assessing the diagnostic value of the measurements was beyond the scope of this study and should be addressed at a later stage of development. Last, the software was compared against a panel of clinicians with different professions, as the diagnosis and treatment of carpal instability often involves a multidisciplinary approach. Although this comparison provided an estimate of the performance across clinicians, the heterogeneity of the panel also meant that no universal performance could be extracted.

In conclusion, this study provides preliminary evidence that an automated AI system can accurately measure and detect radiological signs of carpal instability. The automated measurements and detections were found to be largely comparable to those of clinicians and may help to raise awareness of carpal instability in clinical practice. The system displayed the potential in improving the detections of carpal arc interruptions by both specialized and less specialized clinicians. The proposed framework could be useful for automating other carpal instability measurements and measurements in other musculoskeletal structures. Future research should validate the AI system in an observer study with more clinicians per profession and investigate its potential impact on patient outcomes in a concurrent reading setting.

## Supplementary information

Electronic Supplementary Material

## Abbreviations

AI: Artificial intelligence
AP: Anterior-posterior
CL: Capitolunate
LoA: Limits of agreement
MAE: Mean absolute error
MFD: Mean Fréchet distance
MSK: Musculoskeletal
PA: Posterior-anterior
SL: Scapholunate
