# Supplementary material for: Artificial intelligence for automated detection and measurements of carpal instability signs on conventional radiographs
Source: Eur Radiol. 2024 Apr 18;34(10):6600–13. doi: 10.1007/s00330-024-10744-1 (PMC11399222; doi:10.1007/s00330-024-10744-1)
Supplement: Supplementary file 1 — Electronic Supplementary Material [file 330_2024_10744_MOESM1_ESM.pdf]

Artificial Intelligence for Automated Detection and Measurements of Carpal Instability Signs on Conventional Radiographs

Electronic Supplementary Material (ESM)

Eur Radiol (2024) Hendrix N, Hendrix W, Maresch B et al.

Appendix E1. Additional Imaging Parameters for Table 1

Table E1 shows an overview of additional imaging parameters for the radiographs in the experimental datasets to complement the parameters provided in Table 1 in the main text.

Appendix E2. Annotation Protocol for Dataset 1

Dataset 1 was annotated using the software Cirrus Core Workstation on the web platform Grand Challenge (version 2022.07, 2022) [1] using the following protocol. For developing the segmentation component in the artificial intelligence (AI) pipeline, medical students J.D. and K.R. first segmented either the anterior side (frontal view, including [neutral, ulnar-deviated, clenched fist] anterior-posterior [AP] or posterior-anterior [PA] and oblique view) or lateral side (lateral view) of the following carpal bones in each radiograph: scaphoid, lunate, capitate, hamate, and triquetrum (latter two bones on frontal view only). Next, the author N.H. verified and refined all segmentation masks and consulted with an experienced MSK radiologist (M.R.) in case of doubt. In a random subset of 200 radiographs (100 frontal view, 100 lateral view) that was reserved for validation (see training procedure in Appendix E7), N.H. also measured the scapholunate (SL) joint distance in millimeter and the SL and capitolunate (CL) angle.

For developing the landmark localization component, N.H. marked the articular facet surfaces of these bones with polylines (i.e., lists of points with straight line segments drawn between consecutive points) in a random subset of 400 radiographs stratified by radiographic view (frontal or lateral view) and hospital. On frontal view radiographs, all articular surfaces between the selected bones were annotated. For the bones in the proximal row, the facet surfaces on the radius and ulna (i.e., radiocarpal joint) were also annotated. The surface of the lunate articulating with the hamate was only considered part of the facet surface in case of a lunate type II morphology, as defined by Viegas et al. [2]. The articular surfaces between the triquetrum and hamate were considered part of the carpal arcs until they started to bend when moving into the ulnar direction, and the articular surfaces beyond that point were separately annotated. On lateral view radiographs, the articular

surfaces of the following facets were annotated: *(a)* the third metacarpal and lunate facet of the capitate, *(b)* the capitate and radius facets of the lunate.

For developing the carpal arc interruption detection component, N.H. labelled for each frontal view radiograph whether any signs of carpal instability were present according to the original radiology reports. Then, polylines of the carpal arcs were generated for radiographs without any reported signs of carpal instability using the developed AI pipeline. This way, a shape model of non-interrupted carpal arcs could be built (see Appendix E7). Finally, the generated carpal arcs were visually inspected by N.H. to check for any significant inaccuracies in either the generated carpal arcs or derived carpal stability status. This resulted into a selection of 511 radiographs (434 AP/PA view, 77 oblique view) without carpal instability.

Appendix E3. Training Data Selection

Figure E1 shows a flowchart of the radiograph selection for the training dataset (dataset 1). The sample size was determined based on the data required to saturate the performance of the models. To maximize the variance in the dataset, only one frontal view (including [neutral, ulnar-deviated, clenched fist] AP/PA, and oblique view) and one lateral view radiograph was sampled per patient. Finger series were excluded during the data selection, as they provide little coverage of the carpal bones and are not suited for conducting carpal instability measurements.

Appendix E4. Measurement Value Distribution of Dataset 2

Figure E2 shows a histogram of the distribution of the SL distance, SL angle, and CL angle measurements for the dataset 2 and the observer study subset.

Appendix E5. Consensus Reading Procedure

The test dataset (datasets 2) was independently annotated by two musculoskeletal (MSK) radiologists (see main text). The following annotations per patient were created: *(a)* SL joint distance in millimeter, *(b)* continuous lines of the carpal arcs (i.e., polylines), *(c)* binary label indicating whether the carpal arcs are interrupted, *(d)* presence of type II lunate morphology or shortened triquetrum, *(e)* the SL and CL angle in degrees (automatically calculated from axes). The radiologists had access to all radiographic views per patient and assessed the cases using the software Cirrus Core Workstation on the web platform Grand Challenge (version 2022.07, 2022) [1].

After this procedure, any discrepant measurements were identified by applying the interquartile range (IQR) method [3]. The interquartile range is the difference between the first quartile (Q1) and third quartile (Q3), and measurement values outside 1.5 × IQR below Q1 or 1.5 × IQR above Q3 were considered outliers. The similarity between the polylines of the carpal arcs was quantified and compared using the Fréchet distance metric [4]. The Fréchet distance can be explained as the minimum cord-length required to able to transverse two lines. If no discrepancies were found, then the measurement values of the radiologists were combined by calculating the average. In case of the carpal arc polylines, the polylines were uniformly resampled on 300 points and then the coordinates were averaged.

Appendix E6. Image Processing Steps

The following steps were taken for processing a given image in the AI pipeline:

1. *Pre-process image*:
    a. Rescale pixel data to a 16-bit intensity range.
    b. Normalize the pixel spacing of the image using the Pixel (Imager) Spacing attribute from the original Digital Imaging and Communications in Medicine (DICOM) file. Image shrinking and enlarging are respectively conducted using the "INTER_AREA" (i.e., resampling using pixel area relation) and "INTER_CUBIC" (i.e., bicubic

interpolation) method from the OpenCV Python library (version 4.7.0.68, 2022) [5]. Save a copy of the original image.

c. Fix the image size to 1600 × 1600 pixels by zero padding or center-cropping.

2. *Extract regions-of-interest*:

a. If a frontal view radiograph is provided, segment the anterior side of the scaphoid, lunate, triquetrum, capitate, and hamate with a segmentation convolutional neural network (CNN) optimized for processing frontal view radiographs. If a lateral view radiograph is provided, segment the lateral side of the scaphoid, lunate, and capitate with another segmentation CNN optimized for processing lateral view radiographs. The architecture and training procedure of the CNNs are described in Appendix E7. The detection threshold was set to 0.5.

b. Smooth the segmentation masks by applying morphological closing with either a 13 × 13 pixel (frontal view) or 15 × 15 pixel (lateral view) disk kernel. A disk kernel is chosen over a standard square kernel to better preserve the organic shapes of the bones.

c. Compute the number of connected components for each segmentation mask and remove all but the largest connected component. This step removes noise and leaves only the largest segmented object, which is expected to be the bone mask.

d. Project the segmentation masks back onto the original input image by reversing the normalization operations conducted in the previous steps.

e. Extract a 33 × 33 millimeter patch surrounding each segmented bone in the original image and mask. To ensure that all bones fit within the patch, the patch size was set to the 99th percentile bounding box width or height of the segmented bones in dataset 1. The image and mask are cropped to a fixed-size patch instead of a bounding box of the segmentation mask to preserve the scale. The angle of rotation of the bone is aligned to either the minor axis (lunate) or major axis (other bones)

before extracting the patch (orientation is estimated through ellipse fitting on mask). A patch with the original bone orientation is also saved.

f. Standardize and enhance the image contrast of each patch using the contrast stretching method. Use the minimum and maximum value in the bounding box of the segmentation mask in the original image as output value range.

g. Standardize the laterality (left or right hand) by processing the scaphoid patch with the laterality detection CNN from Hendrix et al. [6] and flipping the extracted patches if the radiograph depicts a left hand. For compatibility purposes, the scaphoid patch is resized to 299 × 299 pixels using bilinear interpolation and the patch with the original bone orientation is used as input. The pixel values are normalized by first rescaling the values between 0 and 1 via min-max scaling and then zero centering the values using the per-channel mean and standard deviation of the ImageNet dataset [7]. The laterality is standardized to improve the landmark fitting results of the active appearance models (AAMs) in the next step (see Appendix E7 for more details). The laterality is determined visually instead of using the metadata of the DICOM file, as this information is not always available in the metadata.

3. *Fit landmarks to articular facet joint surfaces*:

   a. For each segmented bone (with exception of the scaphoid on the lateral view), localize the anatomical landmarks on the articular facet joint surfaces with patch-based AAMs (see training details in Appendix E7). First, given a certain bone, the bounding box from the segmentation mask is used to align the AAM's reference shape (i.e., mean aligned shape in training data) for initializing the fitting procedure. Next, an AAM is fitted to the mask. Finally, the fitted landmarks are passed to a multi-scale AAM (two scales: half and full image resolution) and are finetuned by fitting this AAM to the image. Following the default settings of the Menpo

framework (version 0.10.0, 2021) [8], the AAMs are fitted to the images and masks using Lucas-Kanade optimization with the Wiberg Inverse-Compositional algorithm [9] for a maximum of 20 iterations (15 and 5 iterations at the lowest and highest scale for the multi-scale AAMs). For the multi-scale AAMs, at the lowest scale, 5 shape components and 30 appearance components are used during the fitting procedure. At the highest scale, 20 shape components and 150 appearance components are used. When no multi-scale features are used, the latter setting is applied.

   b. Project the fitted landmarks on the original input image by reversing the normalization operations conducted in the previous steps.

4. *Conduct measurements*:
   a. Measure the SL joint distance by connecting the midpoints of the lunate facet of the scaphoid and the scaphoid facet of the lunate. To compensate for any inaccuracies in the localization of the articular surfaces, the selection of endpoints of the estimated articular surfaces is optimized before selecting the median (middle) anatomical landmark. First, all possible quadrangles that can be defined between the landmarks of the opposite articular surfaces are determined. Then, both the area and entropy of the angles (squareness) of each quadrangle is calculated. Finally, the endpoints (corners) of the left and right side of the quadrangles are ranked by a weighted average between the quadrangle area (weight=1) and squareness (weight=2) (descending order), and the highest ranked pair of endpoints is selected. This way, a pair of midpoints is selected on the facets with an optimal trade-off between the proximity to the original median anatomical landmark (quadrangle area) and parallelism of the underlying articular surfaces (quadrangle squareness).
   b. Measure the SL angle by first assessing the long axis of the scaphoid through ellipse fitting (major axis) and assessing the midplane axis of the lunate by connecting the

mid-points of the capitate and radius facets of the lunate. The angle is then derived from the axes.

c. Measure the CL angle by first assessing the long axis of the capitate by connecting the mid-points of the third metacarpal and lunate facet of the capitate. The angle is then derived from this axis and the lunate axis (obtained in the previous step).

d. Generate polylines (i.e., lists of points with straight line segments drawn between consecutive points) of the three carpal arcs by connecting the anatomical landmarks corresponding to the relevant facet joint surfaces.

5. *Detect and measure the degree of interruptions in the carpal arcs*:

   a. Uniformly resample the three carpal arc polylines using 100 sampling points per arc.

   b. Reconstruct the expected or hypothetical shape of the carpal arc polylines if non-interrupted. An example of this reconstruction is shown in Figure E3.

   c. Determine the reconstruction error per point between the observed and reconstructed hypothetical normal carpal arcs by calculating the pair-wise Euclidean distance.

   d. Convert the reconstruction errors to z-scores using the mean and standard deviation of the reconstruction errors corresponding to non-interrupted carpal arcs in the training data (dataset 1). Reconstruction errors with a z-score of two or greater are considered significant.

   e. Display the reconstruction errors with vectors and color coding (Fig. E3).

   f. Obtain a single detection score by calculating the percentage of significant reconstruction errors. A percentage of reconstruction errors was chosen over the maximum reconstruction error as detection score, because estimating the interruption magnitude using a single point was found to be susceptible to small non-meaningful inaccuracies in the generated polylines of the carpal arcs.

Appendix E7. Training Procedure

All experiments were conducted on a system with an Nvidia RTX Titan graphics card and Intel Core i9 9900K CPU. The two carpal bone segmentation CNNs for processing frontal and lateral view radiographs were trained on dataset 1 using the PyTorch machine learning framework (version 1.13.1, 2023) [10]. The original DICOM files were first converted to 16-bit PNG files. A random subset of 100 radiographs per radiographic view was used for validation and the rest of the dataset was used for training (no patient overlap). The CNNs were randomly initialized by applying normal initialization following He's method [11] (intermediate layers) and Xavier's method [12] (output layer) for efficient weight optimization. The architecture was adapted from the scaphoid segmentation model from Hendrix et al. [13], which has a light-weight encoder-decoder structure based on the U-Net architecture [14]. Table E2 provides an overview of the adapted architecture and hyperparameter settings. The number of filters per layer was doubled to compensate for increased input variance resulting from the added bones and larger rotation augmentations (later on more about this). The ADAM optimizer [15] ($\beta 1 = 0.9$, $\beta 2 = 0.999$) was used for weight optimization and minimized the categorical cross entropy loss over a single image:

$$\frac{1}{M}\sum_{i=1}^{M}\frac{1}{N}\sum_{j=1}^{N}-y_{i,j}\log\left(p_{i,j}\right)$$

where M is the number of output masks, N is the number of pixels in the image, and $y_{i,j}$ and $p_{i,j}$ are respectively a binary label and probability indicating whether the pixel belongs to the given mask. The initial learning rate was set to 1×10^{-5} and it was reduced to 1×10^{-6} when the training loss did not decrease for 10 epochs. The training process was ended when the validation loss did not decrease for 10 epochs to prevent potential overfitting. Furthermore, the following data augmentations were applied using the Albumentations image augmentation Python library (version 1.3.0, 2022) [16]: horizontal flipping, horizontal and vertical translation (max. factor 0.0625), scaling (max. factor 0.1, "zoom" in/out), rotation (max. 45 degrees, both directions), grid distortion (max. distortion 0.03, five grid cells per side), increased brightness and contrast (max. factor 0.3 and 0.4).

The translation, scaling, and rotation augmentations were applied with 80% probability, whereas the other augmentations were applied with 50% probability.

The AAMs of the articular surfaces were trained on dataset 1 using the Menpo framework (version 0.10.0, 2021) [8]. AAMs are well-investigated statistical deformable models of object shape and appearance (a.k.a. texture) that can be matched to a new image. Due to the constraints imposed by the shape and appearance modelling, relatively few training examples are required to train an AAM. Since anatomical variation of the articular surfaces is limited and creating annotations of these surfaces is challenging and time-intensive, an AAM was chosen over a deep learning-based model for this task. The training procedure and hyperparameter optimalization was conducted as follows.

The polylines of the articular surfaces were first converted to anatomical landmarks by uniformly resampling a fix set of points. The optimal number of sampling points per surface (per bone and view) was determined by plotting the number of points against the Fréchet distance (mean and standard deviation) between the subsampled and original polylines. Based on this analysis, 20 sampling points per facet joint surface were selected, except for the surfaces between the bones in the proximal row. For these surfaces, 15 sampling points were selected instead. The order and orientation (laterality and angle of rotation) of the landmarks per surface was normalized, and then the landmarks were exported with the corresponding images using the PTS file format (i.e., raw landmark points in text file) and 8-bit PNG file format. The angle of rotation was normalized by assessing the minor axis (lunate) or major axis (other bones) based on the segmentation mask through ellipse-fitting.

Next, per carpal bone (except for the scaphoid on the lateral view) two patch-based AAMs were trained on the annotated images. Unlike a holistic appearance representation that covers the entire texture enclosed within the landmarks, a patch appearance representation only covers the texture enclosed within a fixed-size patch sampled on each landmark. A patch representation was chosen over a holistic representation, because only the bone surfaces needed to be modelled and

the texture enclosed by the landmarks has little descriptive value for this task. The first AAM used the bone segmentation mask as appearance representation and fitted the landmarks based on shape information only. To compensate for small segmentation errors and to add context information, the second AAM finetuned the fitted landmarks while using dense SIFT image features [17] as appearance representation (see also Appendix E6). Dense SIFT features were used instead of the raw pixel data because of their rotation and scale invariant properties.

When building the AAMs, the diagonal of the bounding box enclosing the landmarks was normalized to 150 pixels (resizing the shape and image). For the second AAM, the appearance was modelled at two scales, which included the half and full image resolution (lowest and highest scale). At inference time, the fitting procedure moves from the lowest scale to the highest scale for more robust optimization. The patch dimensions for the first AAM were set to 23 × 23 pixels, and they were set to respectively 15 × 15 pixels (lowest scale) and 23 × 23 pixels (highest scale) for the second AAM. The maximum number of shape and appearance components was set to respectively 20 and 150. These settings were adapted from the provided settings in the documentation of the Menpo framework (version 0.10.0, 2021) [8] and were experimentally found to be already optimal for the task at hand.

The point distribution model (PDM) was trained on shapes of non-interrupted carpal arcs obtained from AP/PA view radiographs in dataset 1 using the Menpo framework (version 0.10.0, 2021) [8]. Similar to the development of the AAMs, the polylines of the carpal arcs were first converted to anatomical landmarks by uniformly resampling a fix set of points and then they were exported as PTS files. Per arc, 100 points were resampled on the corresponding polyline. The number of active components was set to keep 95% of the variance in order to remove any noise captured by the last components. In contrast to the carpal bone segmentation CNNs and articular surface AAMs, using oblique view radiographs for building the PDM was not found to have performance benefits in terms of carpal arc interruption detection.

Appendix E8. Evaluation Procedure for Segmentation and Landmark Localization AI Components

The carpal bone segmentation CNNs were evaluated on dataset 1 using five-fold cross-validation (no patient overlap between folds). The landmark localization AAMs were evaluated on a subset of dataset 1 (see Appendix E2) using ten-fold cross-validation (no patient overlap between folds). The evaluation metrics included the mean Dice similarity coefficient (DSC) and mean symmetric Hausdorff distance (HD), which were calculated using the MedPy medical image processing Python library (version 0.4.0) [18]. When evaluating the segmentation CNNs, the pre- and postprocessing steps were applied as described in Appendix E6 (from step 1 to step 2d). When evaluating the landmark localization AAMs, the mean DSC and HD were calculated by converting the fitted shapes to binary masks. Furthermore, the mean final fitting error between the fitted and ground-truth shapes was calculated (resulting from the Lucas-Kanade optimization). The results across the validation folds were pooled before averaging the metrics.

Appendix E9. Abnormal Measurement Detection Results

Table E3 shows the detection performance of the AI system for abnormal SL distances, SL angles, and CL angles with their 95% CIs in dataset 2. Table E4 shows the corresponding detection results of the AI system and clinicians in the observer study set with their 95% CIs and *p* values.

Appendix E10. Carpal Bone Segmentation Results

Table E5 shows the mean DSC and HD (in millimeter) with standard deviation of the segmentation results per carpal bone and per radiographic view obtained through a five-fold cross-validation on dataset 1. Radiographs depicting both hands (0.5% [10/2178]) were excluded from the analysis to calculate the HD. There were nine radiographs (0.4% [9/2168]) that contained a segmentation failure of one or more bones. Three scaphoids were not segmented in frontal view radiographs where the wrist was in cast (*n* = 1), the scaphoid was incompletely depicted (*n* = 1), or the scaphoid was only slightly ossified and developed (*n* = 1, patient was ten years old). Five lunates were not segmented in

frontal view radiographs where the lunate was incompletely depicted (*n* = 4, <50% visible in three radiographs) or where a wrist with severe scaphoid lunate advanced collapse (SLAC) was depicted (*n* = 1). Two triquetrums were not segmented in frontal view radiographs where the triquetrum was incompletely depicted (<50% visible). One lunate was not segmented in a lateral view radiograph where the wrist was in cast and contained osteosynthesis material causing overprojection. Even though the segmentation failure rate was low, the findings underline the importance of providing radiographs to the AI system with complete and non-obstructed depiction of the wrist.

Appendix E11. Anatomical Landmark Localization Results

Table E6 shows the mean (final) fitting error, DSC, and HD (in millimeter) with standard deviation of the anatomical landmark localization results per carpal bone and per radiographic view obtained through a ten-fold cross-validation on a subset of dataset 1. The results are reported with and without the bone orientation normalization and landmark prefitting processing step (i.e., initialize fitting procedure by first fitting an AAM to the segmentation mask, see steps 2e and 3a in Appendix E6). It is important to note that manual segmentation masks were used for all steps in the evaluation procedure (i.e., training, inference, evaluation). The results show that the landmark prefitting step had the most beneficial effect on the performance metrics, which indicates that bone segmentation has added value for localizing the landmarks. The bone orientation normalization step had less effect on the performance metrics, but the effect might be more pronounced in cases of subluxations with angulation. Applying both the orientation normalization and landmark prefitting step led to the best results overall.

Appendix E12. Bland-Altman Plot Analysis of Measurements of AI and Clinicians

Figures E4-E9 show a Bland-Altman plot with 95% CI bands comparing the measurements of the AI system and clinicians with the reference standard for the SL distances, SL angles, and CL angles in the observer study subset.

References

1. Meakin J, Gerke PK, Kerkstra S, et al (2021) Grand-Challenge.org. Zenodo. DOI:10.5281/ZENODO.6819122.

2. Viegas SF, Wagner K, Patterson R, Peterson P (1990) Medial (hamate) facet of the lunate. J Hand Surg Am 15(4):564–571. DOI:10.1016/S0363-5023(09)90016-8.

3. Hoaglin DC, Iglewicz B, Tukey JW (1986) Performance of some resistant rules for outlier labeling. J Am Stat Assoc 81(396):991–999. DOI:10.1080/01621459.1986.10478363.

4. Alt H, Godau M (1995) Computing the Fréchet distance between two polygonal curves. Int J Comput Geom Appl 5(01n02):75–91. DOI:10.1142/S0218195995000064.

5. Bradski G (2000) The OpenCV Library. Dr Dobb's J Softw Tools Prof Program 25(11):120-125.

6. Hendrix N, Hendrix W, van Dijke K, et al (2022) Musculoskeletal radiologist-level performance by using deep learning for detection of scaphoid fractures on conventional multi-view radiographs of hand and wrist. Eur Radiol 33(3):1575-1588. DOI:10.1007/s00330-022-09205-4.

7. Deng J, Dong W, Socher R, Li L-J, Li K, Fei-Fei L (2009) Imagenet: A large-scale hierarchical image database. In: 2009 IEEE Conference on Computer Vision and Pattern Recognition (CVPR), Miami, Florida, USA, 20-15 June, 2009 (pp. 248–255). IEEE, New York City (Manhattan), New York, USA.

8. Alabort-i-Medina J, Antonakos E, Booth J, Snape P, Zafeiriou S (2014) Menpo: A comprehensive platform for parametric image alignment and visual deformable models. In: Proceedings of the 22nd ACM international conference on Multimedia (MM), Orlando, Florida, USA, November 3-7, 2014 (pp. 679–682). Association for Computing Machinery, New York City, New York, USA.

9. Papandreou G, Maragos P (2008) Adaptive and constrained algorithms for inverse compositional active appearance model fitting. In: 2008 IEEE Conference on Computer Vision and Pattern Recognition (CVPR), Anchorage, Alaska, USA, 23-28 June, 2008 (pp. 1-8). IEEE, New York City (Manhattan), New York, USA. DOI:10.1109/CVPR.2008.4587540.

10. Paszke A, Gross S, Massa F, et al (2019) PyTorch: An imperative style, high-performance deep learning library. In: Proceedings of the 33rd International Conference on Neural Information Processing Systems (NeurIPS), Vancouver, British Columbia, Canada, December 8-14, 2019 (pp. 8026–8037). Curran Associates, New York City (Red Hook), New York, USA.

11. He K, Zhang X, Ren S, Sun J (2015) Delving deep into rectifiers: Surpassing human-level performance on imagenet classification. In: 2015 IEEE International Conference on Computer Vision (ICCV), Santiago, Chile, December 7-13, 2015 (pp. 1026–1034). IEEE, New York City (Manhattan), New York, USA.

12. Glorot X, Bengio Y (2010) Understanding the difficulty of training deep feedforward neural networks. In: Proceedings of the Thirteenth International Conference on Artificial Intelligence and Statistics (PMLR), Sardinia, Italy, 13-15 May, 2010 (pp. 249–256). JMLR, Cambridge, Massachusetts, USA.

13. Hendrix N, Scholten E, Vernhout B, et al (2021) Development and Validation of a Convolutional Neural Network for Automated Detection of Scaphoid Fractures on Conventional Radiographs. Radiol Artif Intell 3(4):e200260. DOI:10.1148/RYAI.2021200260.

14. Ronneberger O, Fischer P, Brox T (2015) U-net: Convolutional networks for biomedical image segmentation. In: 18th International Conference on Medical Image Computing and Computer Assisted Intervention (MICCAI), Munich, Germany, October 5-9, 2015 (pp. 234–241). Springer, New York City (Manhattan), New York, USA.

15. Kingma DP, Ba J (2015) Adam: A method for stochastic optimization. In: 3rd International Conference on Learning Representations (ICLR), San Diego, California, USA, May 7-9, 2015. ArXiv, Ithaca, New York, USA. DOI:10.48550/arXiv.1412.6980.

16. Buslaev A, Iglovikov VI, Khvedchenya E, Parinov A, Druzhinin M, Kalinin AA (2020) Albumentations: Fast and flexible image augmentations. Information 11(2):125. DOI:10.3390/INFO11020125.

17. Lowe DG (2004) Distinctive image features from scale-invariant keypoints. Int J Comput Vis 60(2):91–110. DOI:10.1023/B:VISI.0000029664.99615.94.

18. Maier O (2019) MedPy 0.4.0. Zenodo. DOI:10.5281/zenodo.2565940.

Tables

Table E1: Additional Imaging Parameters of the Radiographs in the Experimental Datasets

Variable	Dataset 1	Dataset 2 (total)	Dataset 2 (subset)
Image size, pixels			
Hand x-ray height	1641 ± 336	1437 ± 214	1238 ± 394
Hand x-ray width	1103 ± 403	907 ± 179	777 ± 186
Wrist x-ray height	1290 ± 272	1023 ± 199	1065 ± 190
Wrist x-ray width	689 ± 282	564 ± 151	578 ± 161
Scaphoid x-ray height	820 ± 308	1026 ± 275	1214 ± 74
Scaphoid x-ray width	597 ± 214	907 ± 369	1165 ± 153
Pixel size, mm			
Hand x-ray	0.146 ± 0.004	0.16 ± 0	0.16 ± 0
Wrist x-ray	0.146 ± 0.003	0.159 ± 0.004	0.159 ± 0.007
Scaphoid x-ray	0.147 ± 0.003	0.16 ± 0	0.16 ± 0
KVP	47.1 ± 1.86	50.3 ± 1.75	50.8 ± 1.19
Exposure (mAs)	2.75 ± 0.88	2.99 ± 0.08	3.00 ± 0
Model names x-ray devices	DigitalDiagnost (PMS)*<br>Fluorospot Compact FD (SH)*<br>CXDI (CI)<br>DuraDiagnostCompact (PMS)<br>MobileDiagnost wDR (PMS)<br>FD-X (SH)<br>DRX-EVOLUTION (CH)<br>DGR-C76J2O/WR (SE)<br>PCR Eleva (PMS)	CXDI (CI)	CXDI (CI)

Note. — The mean image size, pixel size, KVP, exposure, and patient age are reported with the standard deviation. The model names of the x-ray devices are listed in descending order of frequency: the names marked with an asterisk account for 95% or more of the data. The manufacturer names are abbreviated and are reported in parentheses. Canon Inc. = CI, Carestream Health (CH), KVP = kilovoltage peak, mAs = milliampere-seconds, mm = millimeter, PMS = Philips Medical Systems, Samsung Electronics = SE, Siemens Healthineers = SH.

Table E2: Overview of the Segmentation Network Architecture and Hyperparameter Settings

Encoder layer	Activation	Output shape (channels × height × width)	Hyperparameter settings
Input image	-	1 × 1600 × 1600	-
Convolution	LReLU	32 × 800 × 800	kernel size = 4, stride = 2, padding = 1
Convolution	LReLU	64 × 400 × 400	kernel size = 4, stride = 2, padding = 1
Convolution	LReLU	128 × 200 × 200	kernel size = 4, stride = 2, padding = 1
Convolution	LReLU	256 × 100 × 100	kernel size = 4, stride = 2, padding = 1
Convolution	LReLU	512 × 50 × 50	kernel size = 4, stride = 2, padding = 1
Convolution	LReLU	1024 × 25 × 25	kernel size = 4, stride = 2, padding = 1
**Decoder layer**	**Activation**	**Output shape (channels × height × width)**	**Hyperparameter settings**
Latent variable	-	1024 × 25 × 25	-
Upsample	-	1024 × 50 ×50	upscale factor = 2, mode = 'nearest'
Convolution	LReLU	512 × 50 ×50	kernel size = 5, stride = 1, padding = 2
Upsample	-	512 × 100 × 100	upscale factor = 2, mode = 'nearest'
Convolution	LReLU	256 × 100 × 100	kernel size = 5, stride = 1, padding = 2
Upsample	-	256 × 200 × 200	upscale factor = 2, mode = 'nearest'
Convolution	LReLU	128 × 200 × 200	kernel size = 5, stride = 1, padding = 2
Upsample	-	128 × 400 × 400	upscale factor = 2, mode = 'nearest'
Convolution	LReLU	64 × 400 × 400	kernel size = 5, stride = 1, padding = 2
Upsample	-	64 × 800 × 800	upscale factor = 2, mode = 'nearest'
Convolution	LReLU	32 × 800 × 800	kernel size = 5, stride = 1, padding = 2
Upsample	-	32 × 1600 ×1600	upscale factor = 2, mode = 'nearest'
Convolution	Sigmoid	5 × 1600 ×1600	kernel size = 5, stride = 1, padding = 2

Note. — Zero-padding was added to each side of the input. The angle of the negative slope of the leaky rectified linear unit (LReLU) activation function was set to 0.2. Skip connections (i.e., channel wise concatenations) were added between the encoder and decoder layers to retain spatial information throughout the network. Each channel in the final output represents a separate mask per class (i.e., scaphoid, lunate, triquetrum, capitate, hamate).

Table E3: Abnormal Measurement Detection Results of the AI System on Dataset 2

	SL distance abnormality detection	SL angle abnormality detection	CL angle abnormality detection
Sensitivity (%)			
Value	64 (51, 70)	84 (76, 91)	70 (50, 90)
Proportion	49/77	82/98	14/20
Specificity (%)			
Value	98 (93, 98)	74 (65, 83)	95 (92, 98)
Proportion	178/181	69/93	163/171

Note. —95% confidence intervals are reported in parentheses. The abnormality thresholds for the measurements are defined in the main text. CL = capitolunate, SL = scapholunate.

Table E4: Comparison of Abnormal Measurement Detection Results between the AI System and Clinicians

	Abnormal SL distance detection (n_{total} = 87, n_{pos} = 39, n_{neg} = 48)					
	Sensitivity (%)			Specificity (%)		
Reader	Value	Frac	*p*	Value	Frac	*p*
AI	72 (56, 85)	28/39		98 (94, 100)	47/48	
Junior doctor	80 (67, 92)	31/39	.46	92 (83, 98)	44/48	.39
Hand surgeon	92 (82, 100)	36/39	.01	92 (83, 98)	44/48	.25
ER doctor	100 (97, 100)	39/39	.002	81 (69, 92)	39/48	.01
Radiologist	87 (74, 97)	34/39	.07	90 (81, 98)	43/48	.24
MSK radiologist	90 (80, 97)	35/39	.06	92 (83, 98)	44/48	.22
	Abnormal SL angle detection (n_{total} = 87, n_{pos} = 38, n_{neg} = 49)					
	Sensitivity (%)			Specificity (%)		
Reader	Value	Frac	*p*	Value	Frac	*p*
AI	90 (79, 97)	34/38		71 (59, 84)	35/49	
Junior doctor	92 (82, 100)	35/38	>.99	63 (49, 76)	31/49	.49
Hand surgeon	82 (68, 92)	31/38	.52	80 (69, 90)	39/49	.50
ER doctor	66 (53, 82)	25/38	.03	88 (78, 96)	43/49	.12
Radiologist	95 (87, 100)	36/38	.64	65 (53, 80)	32/49	.58
MSK radiologist	84 (71, 95)	32/38	.73	84 (74, 94)	41/49	.19
	Abnormal CL angle detection (n_{total} = 87, n_{pos} = 20, n_{neg} = 67)					
	Sensitivity (%)			Specificity (%)		
Reader	Value	Frac	*p*	Value	Frac	*p*
AI	70 (50, 90)	14/20		97 (93, 100)	65/67	
Junior doctor	70 (45, 90)	14/20	>.99	99 (96, 100)	66/67	>.99
Hand surgeon	75 (55, 95)	15/20	>.99	97 (93, 100)	65/67	>.99
ER doctor	65 (45, 85)	13/20	>.99	97 (93, 100)	65/67	>.99
Radiologist	85 (70, 100)	17/20	.51	99 (96, 100)	66/67	>.99
MSK radiologist	95 (85, 100)	19/20	.07	99 (96, 100)	66/67	>.99

Note. —95% confidence intervals are reported in parentheses. The *p* values refer to the differences in evaluation metrics with respect to the artificial intelligence (AI) system. “Fraction” has been abbreviated to “Frac”. CL = capitolunate, ER = emergency room, MSK = musculoskeletal, SL = scapholunate.

Table E5: Carpal Bone Segmentation Results on Dataset 1

Bone	*n*	Radiographic view	Mean DSC	Mean HD (mm)
Scaphoid	1,103	Frontal	0.95 ± 0.09	1.5 ± 1.7
Lunate	1,095	Frontal	0.94 ± 0.12	1.3 ± 1.3
Triquetrum	1,089	Frontal	0.90 ± 0.14	1.7 ± 1.1
Capitate	1,107	Frontal	0.95 ± 0.04	2.0 ± 1.3
Hamate	1,098	Frontal	0.90 ± 0.12	2.0 ± 1.5
Scaphoid	1,060	Lateral	0.93 ± 0.06	2.4 ± 1.8
Lunate	1,060	Lateral	0.93 ± 0.09	1.8 ± 1.7
Capitate	1,061	Lateral	0.94 ± 0.04	2.2 ± 1.3

Note. — The metrics are reported with their standard deviation. Each bone is not always depicted and therefore the number of masks (*n*) is reported. Frontal view included neutral, ulnar-deviated, clenched fist anterior-posterior (AP) or posterior-anterior (PA) view and oblique view. DSC = Dice similarity coefficient, HD = symmetric Hausdorff distance, mm = millimeter.

Table E6: Anatomical Landmark Localization Results per Bone on Dataset 1

Bone	Orientation normalization	Prefitting	Radiographic view	Mean fitting error	Mean DSC	Mean HD (mm)
Scaphoid			Frontal	0.047 ± 0.037	0.85 ± 0.08	3.6 ± 1.8
Lunate			Frontal	0.062 ± 0.052	0.86 ± 0.07	3.0 ± 1.9
Triquetrum			Frontal	0.073 ± 0.049	0.79 ± 0.11	**3.3 ± 1.5**
Capitate			Frontal	0.058 ± 0.052	0.87 ± 0.09	4.0 ± 2.5
Hamate			Frontal	0.075 ± 0.061	0.66 ± 0.15	4.7 ± 2.7
Lunate			Lateral	0.081 ± 0.051	0.84 ± 0.07	4.8 ± 2.9
Capitate			Lateral	0.060 ± 0.048	0.85 ± 0.09	4.2 ± 2.8
Scaphoid		✓	Frontal	**0.042 ± 0.024**	**0.86 ± 0.07**	3.4 ± 1.7
Lunate		✓	Frontal	**0.052 ± 0.041**	**0.87 ± 0.06**	**2.4 ± 1.1**
Triquetrum		✓	Frontal	**0.068 ± 0.042**	**0.81 ± 0.09**	3.3 ± 1.6
Capitate		✓	Frontal	0.048 ± 0.032	**0.89 ± 0.05**	**3.5 ± 1.6**
Hamate		✓	Frontal	0.055 ± 0.036	0.72 ± 0.10	3.6 ± 1.6
Lunate		✓	Lateral	0.063 ± 0.031	0.85 ± 0.06	4.0 ± 2.2
Capitate		✓	Lateral	0.048 ± 0.033	0.88 ± 0.06	3.6 ± 2.3
Scaphoid	✓		Frontal	0.047 ± 0.028	0.85 ± 0.08	3.4 ± 1.6
Lunate	✓		Frontal	0.082 ± 0.091	0.85 ± 0.08	3.0 ± 1.8
Triquetrum	✓		Frontal	0.074 ± 0.045	0.80 ± 0.11	**3.3 ± 1.5**
Capitate	✓		Frontal	0.049 ± 0.029	0.88 ± 0.05	3.7 ± 2.0
Hamate	✓		Frontal	0.068 ± 0.050	0.67 ± 0.14	4.1 ± 2.0
Lunate	✓		Lateral	**0.055 ± 0.023**	**0.86 ± 0.06**	**3.5 ± 2.0**
Capitate	✓		Lateral	0.047 ± 0.022	0.88 ± 0.05	3.4 ± 1.6
Scaphoid	✓	✓	Frontal	0.044 ± 0.022	**0.86 ± 0.07**	**3.2 ± 1.6**
Lunate	✓	✓	Frontal	0.081 ± 0.096	0.85 ± 0.08	2.8 ± 1.4
Triquetrum	✓	✓	Frontal	0.069 ± 0.039	**0.81 ± 0.09**	3.3 ± 1.6
Capitate	✓	✓	Frontal	**0.047 ± 0.026**	**0.89 ± 0.05**	3.8 ± 2.4
Hamate	✓	✓	Frontal	**0.054 ± 0.034**	**0.73 ± 0.09**	**3.3 ± 1.4**
Lunate	✓	✓	Lateral	**0.055 ± 0.023**	**0.86 ± 0.06**	3.6 ± 2.1
Capitate	✓	✓	Lateral	**0.046 ± 0.019**	**0.89 ± 0.05**	**3.3 ± 1.4**

Note. — The articular facet joint surfaces were labelled in 400 radiographs from dataset 1 (200 frontal view, 200 lateral view, equally distributed between hospitals). All bones were (fully) depicted in the radiographs. The metrics are reported with their standard deviation. Per bone and radiographic view, metrics with the most optimal values across the configurations are made in bold (lowest fitting error [output cost function Wiberg Inverse-Compositional algorithm], lowest Hausdorff distance [HD], and highest Dice similarity coefficient [DSC]; lowest standard deviation in case of equal values). "Prefitting" refers to whether the fitting procedure was initialized by prefitting an active appearance model to the segmentation masks (see Appendices E6 and E7). Frontal view included neutral, ulnar-deviated, clenched fist anterior-posterior (AP) or posterior-anterior (PA) view and oblique view. DSC = Dice similarity coefficient, HD = symmetric Hausdorff distance, mm = millimeter.

Figures

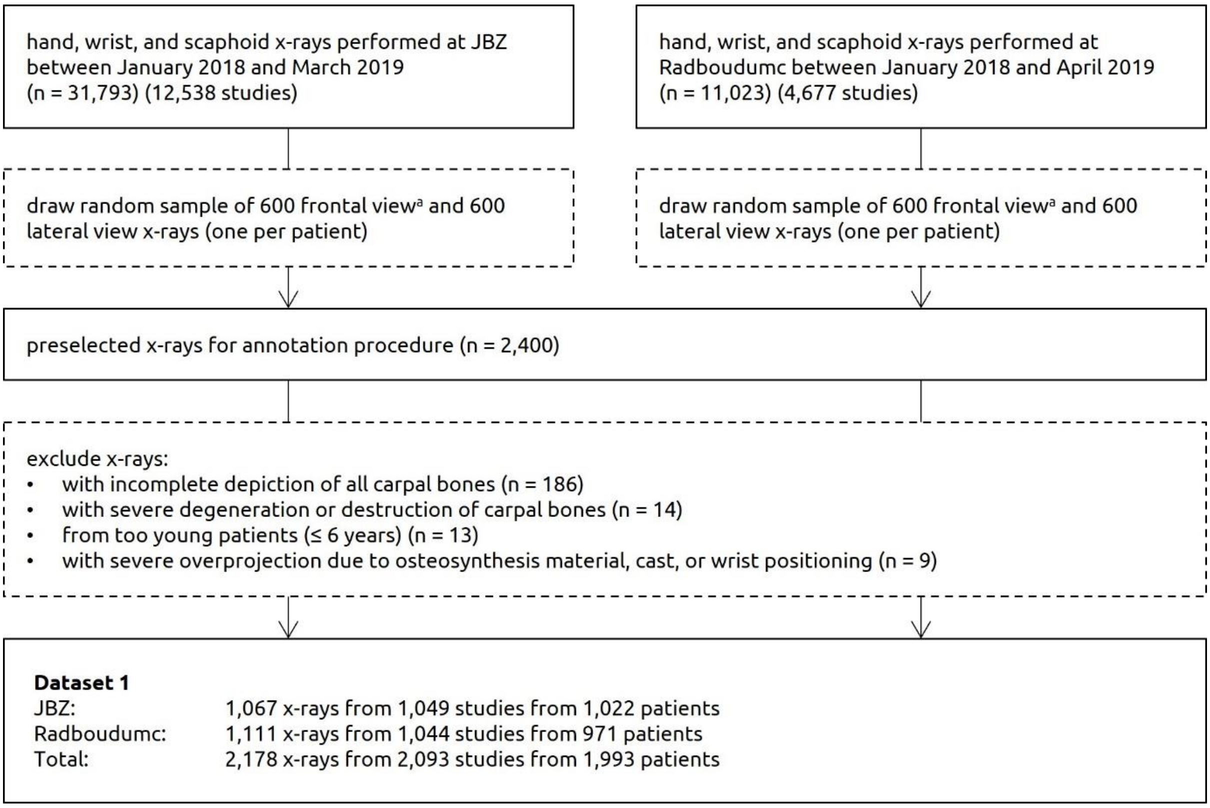

Figure E1. Flowchart for the inclusion and exclusion of x-rays in dataset 1 (training). The number of x-rays at each step is denoted with *n*. JBZ = Jeroen Bosch Hospital, Radboudumc = Radboud University Medical Center.

[a] frontal view radiographs included neutral, ulnar-deviated, and clenched fist anterior-posterior [AP] or posterior-anterior [PA] and oblique radiographs.

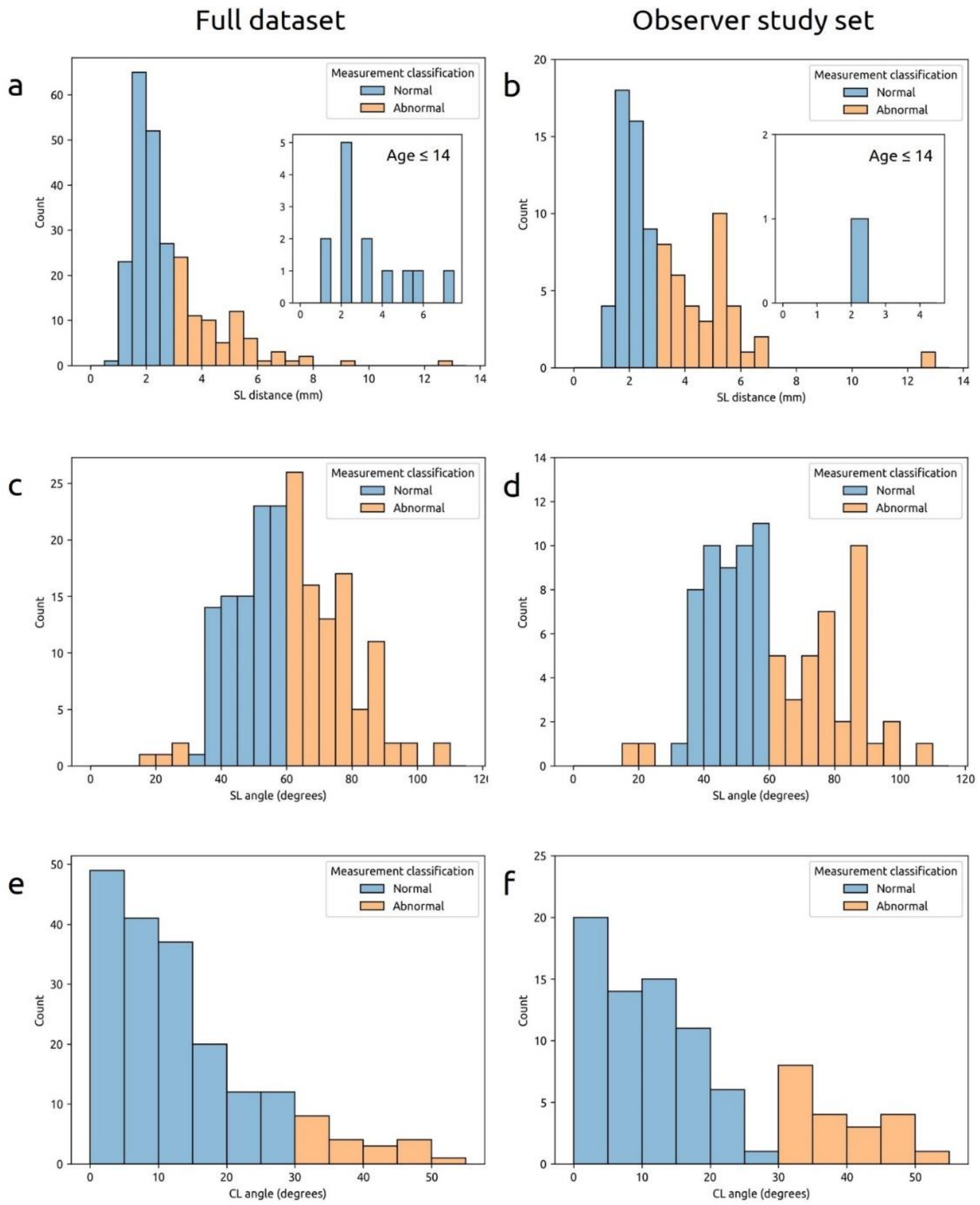

Figure E2. Histograms of the measurement value distributions of the scapholunate (SL) distance, SL angle, and capitolunate (CL) measurements in dataset 2 and the observer study subset (**a**-**f**). The bars for the distance and angle measurements are respectively plotted per 0.5 mm and 5 degrees. The distinction between normal and abnormal measurement values is color-coded (see legends). It is important to note that different SL distance cut-off points were selected for children (≤ 14 years), and these patients are represented in embedded subplots. **a**-**b** SL distance distribution for the full dataset (left, *n* = 258) and subset (right, *n* = 87). **c**-**d** SL angle distribution for the full dataset (left, *n* = 189) and subset (right, *n* = 87). **e**-**f** CL angle distribution for the full dataset (left, *n* = 191) and subset (right, *n* = 87).

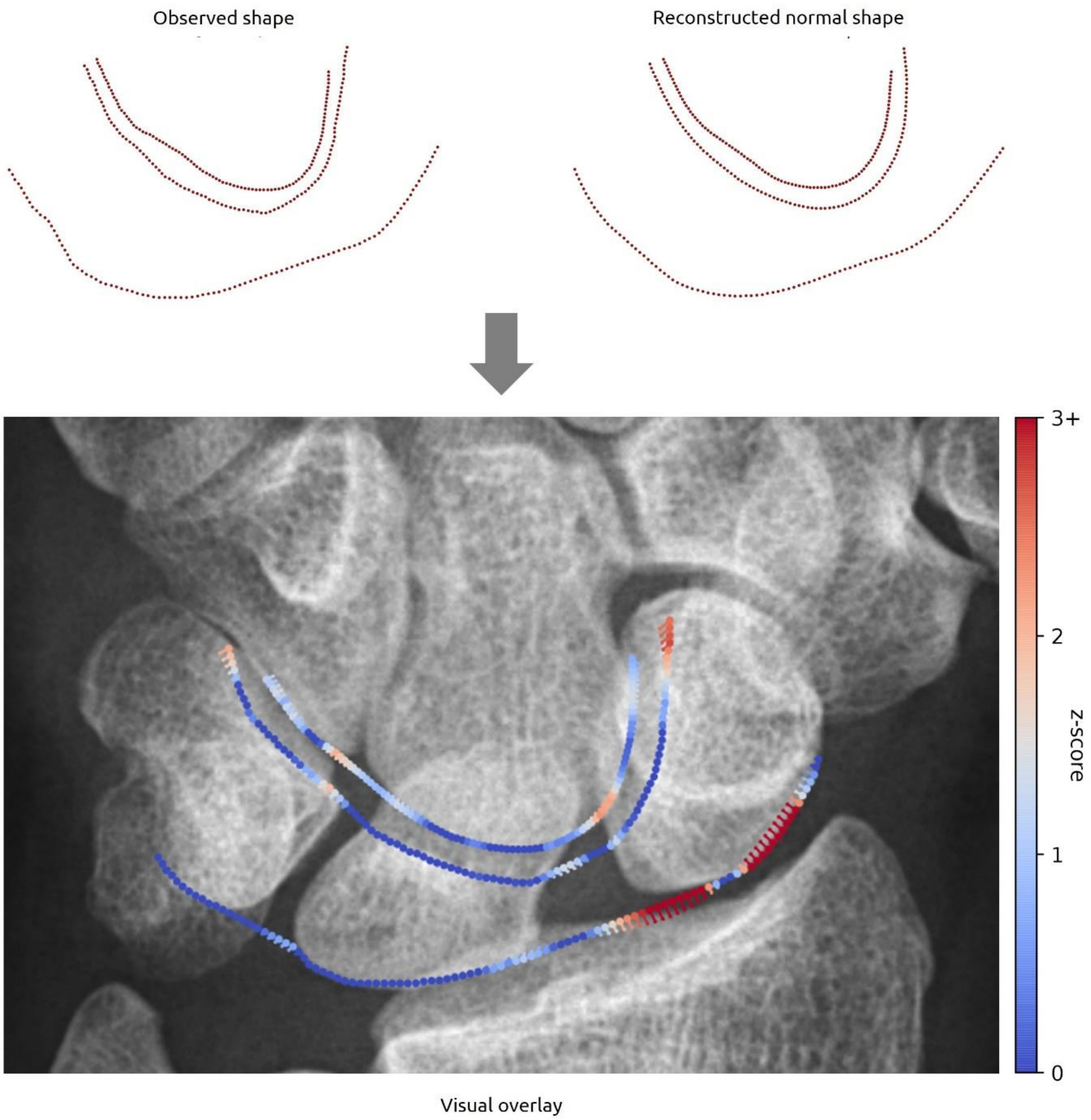

Figure E3. Example of carpal arcs detected by the artificial intelligence (AI) system (top left) and its reconstructed hypothetical normal shape (top right). The reconstruction error per point is determined by calculating the pair-wise Euclidean distance and is then converted to a z-score. The z-scores are overlaid on the original image as color-coded points on the detected arcs (bottom), where the small, attached tails (displacement vectors) show the distances or deviations from the hypothetical normal shape.

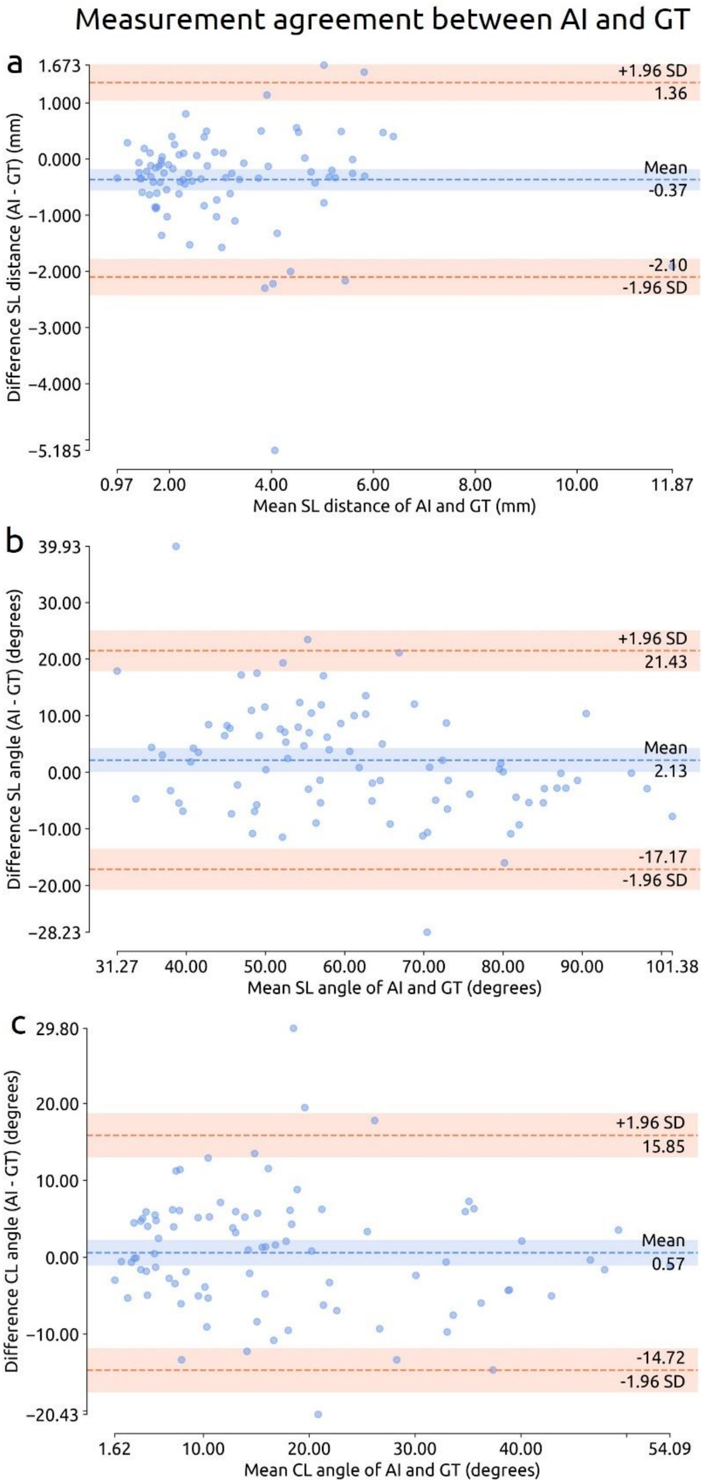

Figure E4. Bland-Altman plots of the measurement agreement between the AI system and the ground-truth (GT) on the scapholunate (SL) distance (**a**), SL angle (**b**), and capitolunate (CL) angle (**c**) measurements in the observer study subset (*n* = 87). Each marker represents one paired measurement. The dashed lines represent the mean difference (blue) and limits of agreement (orange). The shaded bands represent 95% confidence intervals. SD = standard deviation.

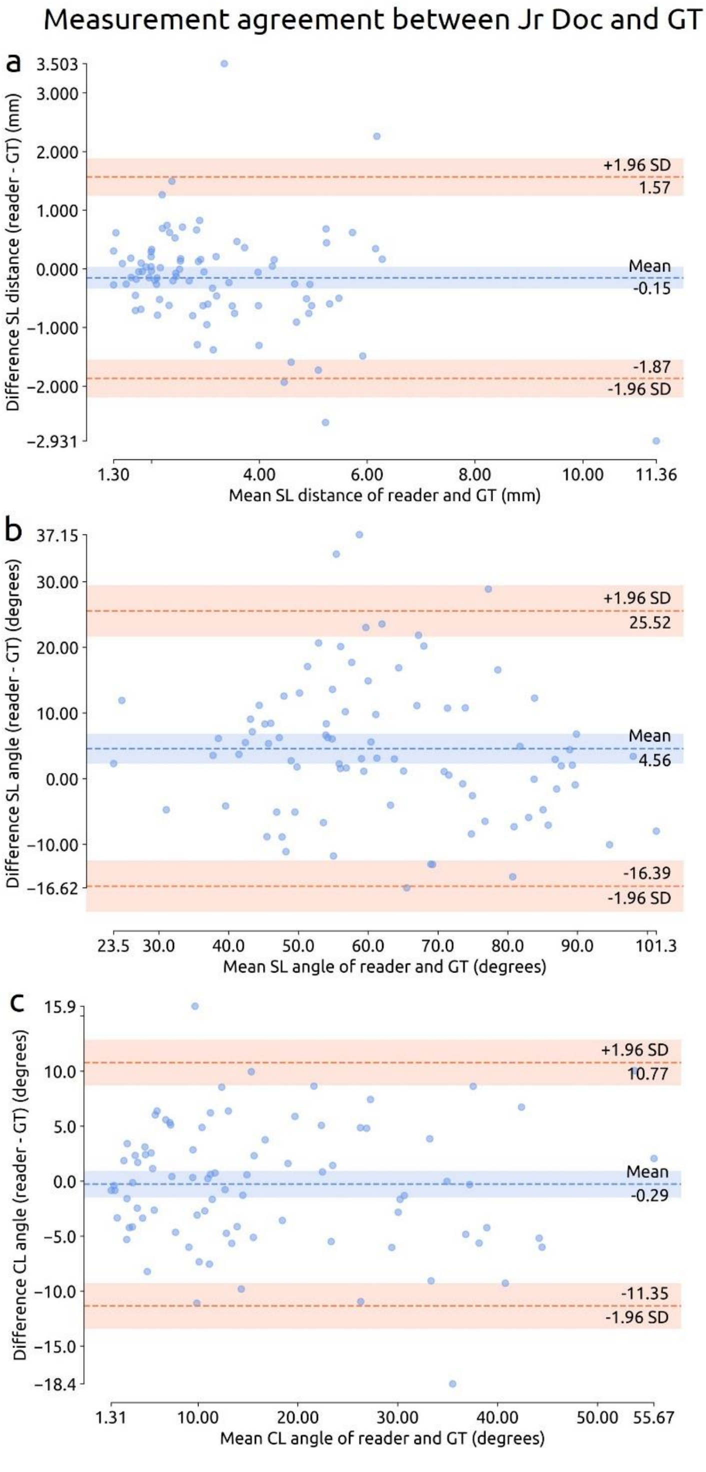

Figure E5. Bland-Altman plots of the measurement agreement between the junior doctor (Jr Doc) and the ground-truth (GT) on the scapholunate (SL) distance (**a**), SL angle (**b**), and capitolunate (CL) angle (**c**) measurements in the observer study subset (*n* = 87). Each marker represents one paired measurement. The dashed lines represent the mean difference (blue) and limits of agreement (orange). The shaded bands represent 95% confidence intervals. SD = standard deviation.

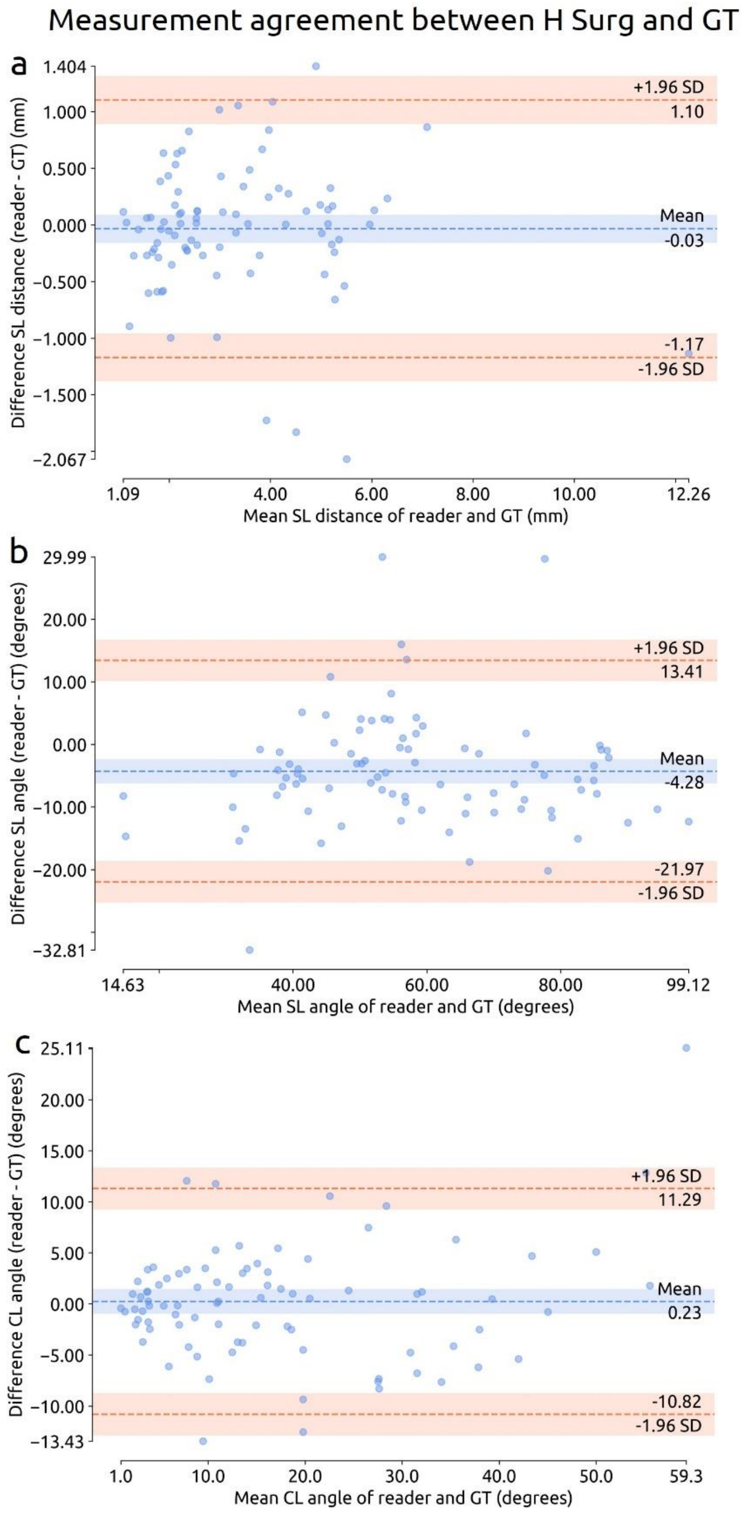

Figure E6. Bland-Altman plots of the measurement agreement between the hand surgeon (H Surg) and the ground-truth (GT) on the scapholunate (SL) distance (**a**), SL angle (**b**), and capitolunate (CL) angle (**c**) measurements in the observer study subset (*n* = 87). Each marker represents one paired measurement. The dashed lines represent the mean difference (blue) and limits of agreement (orange). The shaded bands represent 95% confidence intervals. SD = standard deviation.

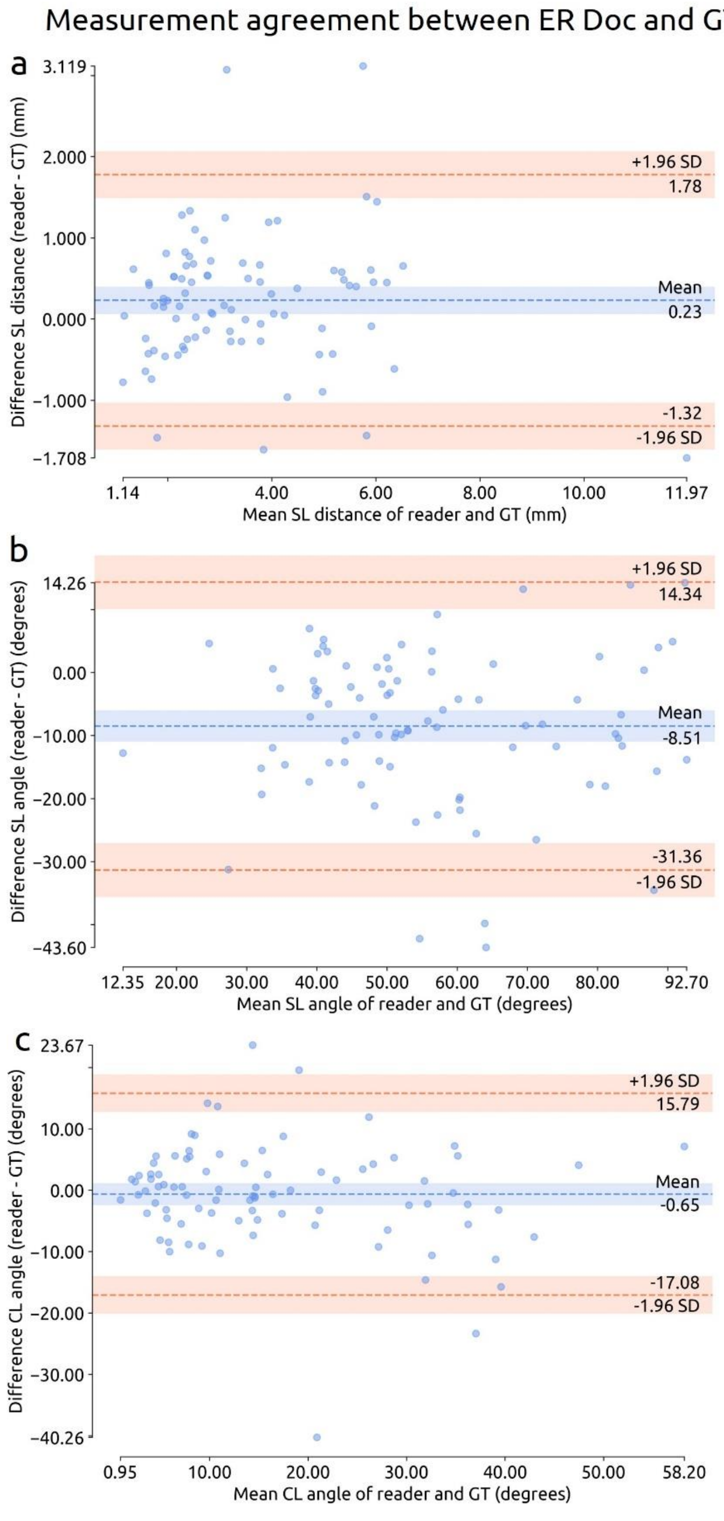

Figure E7. Bland-Altman plots of the measurement agreement between the emergency doctor (ER Doc) and the ground-truth (GT) on the scapholunate (SL) distance (**a**), SL angle (**b**), and capitolunate (CL) angle (**c**) measurements in the observer study subset (*n* = 87). Each marker represents one paired measurement. The dashed lines represent the mean difference (blue) and limits of agreement (orange). The shaded bands represent 95% confidence intervals. SD = standard deviation.

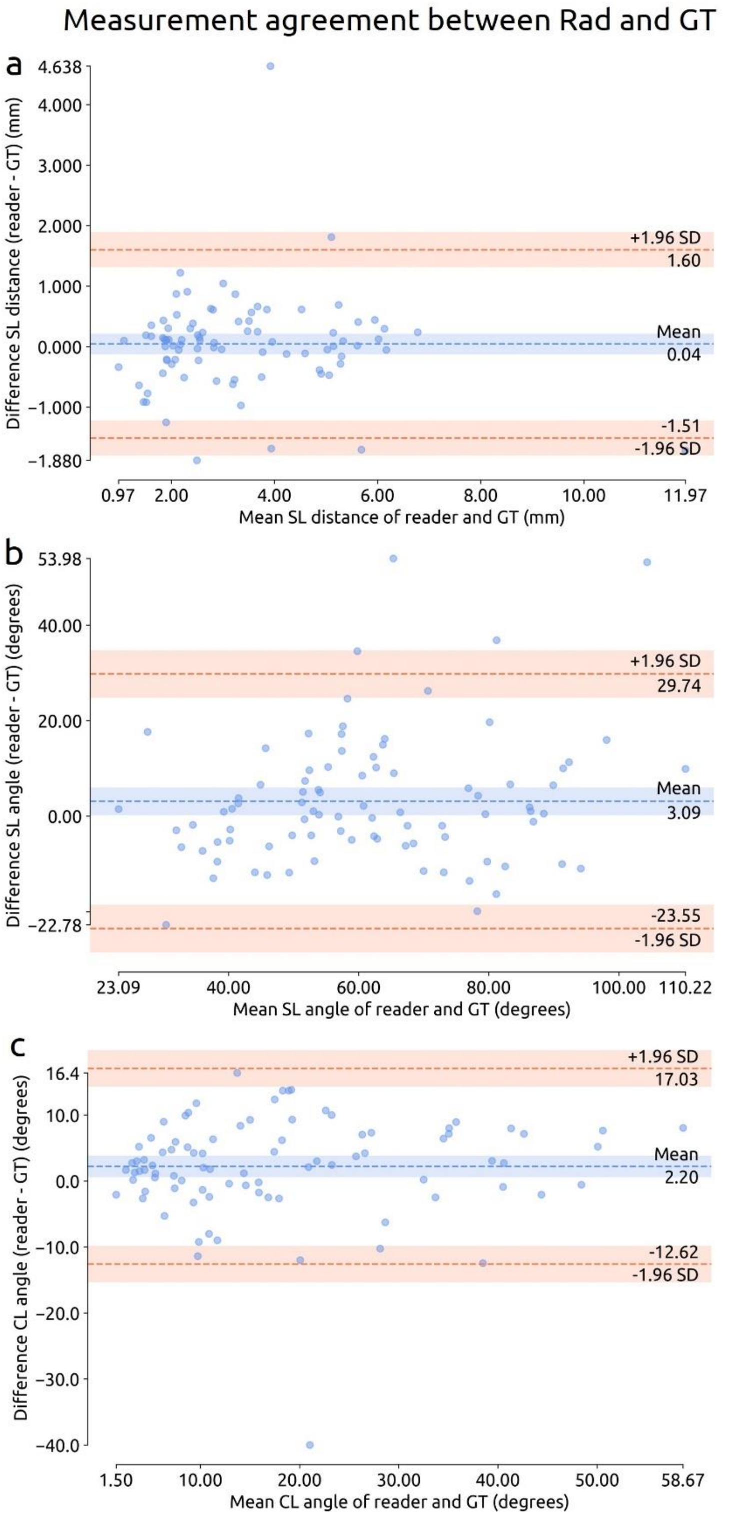

Figure E8. Bland-Altman plots of the measurement agreement between the radiologist (Rad) and the ground-truth (GT) on the scapholunate (SL) distance (**a**), SL angle (**b**), and capitolunate (CL) angle (**c**) measurements in the observer study subset (*n* = 87). Each marker represents one paired measurement. The dashed lines represent the mean difference (blue) and limits of agreement (orange). The shaded bands represent 95% confidence intervals. SD = standard deviation.

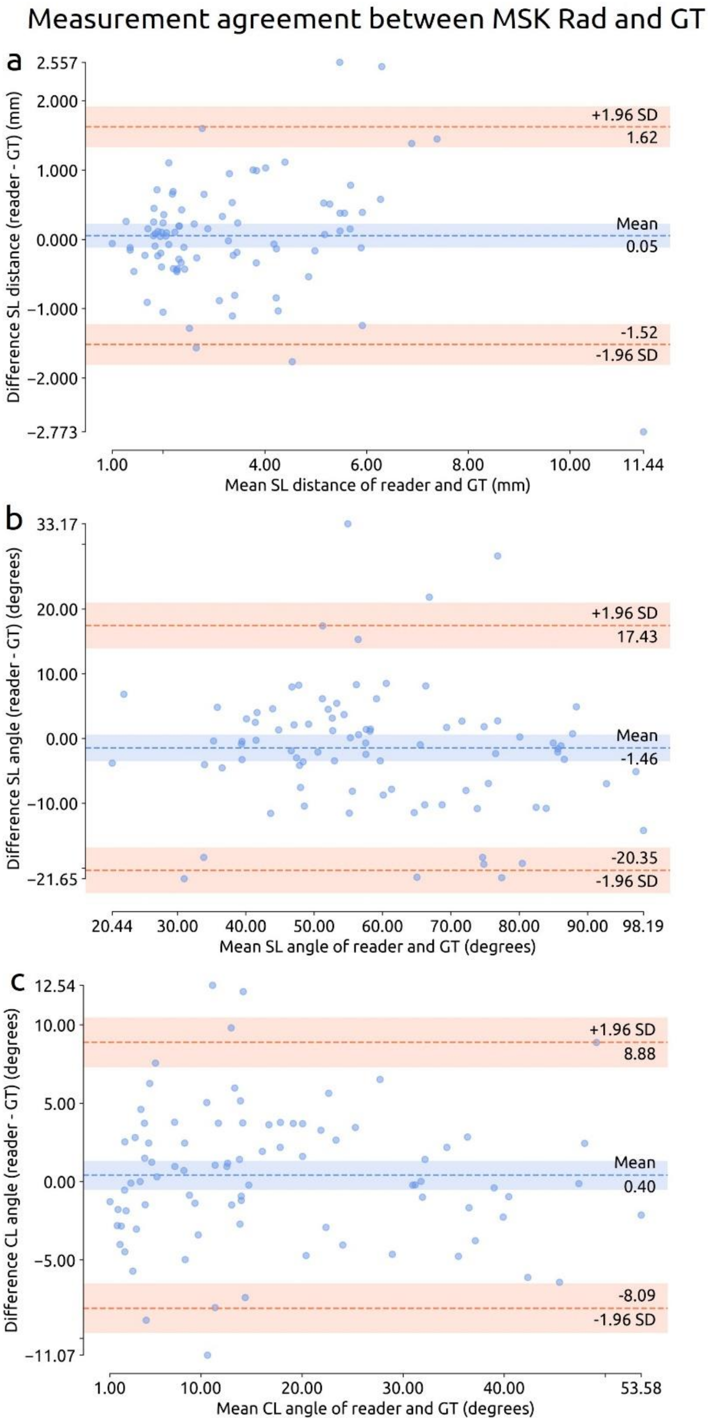

Figure E9. Bland-Altman plots of the measurement agreement between the musculoskeletal radiologist (MSK Rad) and the ground-truth (GT) on the scapholunate (SL) distance (**a**), SL angle (**b**), and capitolunate (CL) angle (**c**) measurements in the observer study subset (*n* = 87). Each marker represents one paired measurement. The dashed lines represent the mean difference (blue) and limits of agreement (orange). The shaded bands represent 95% confidence intervals. SD = standard deviation.
